# Supplementary material for: Projecting heat-related excess mortality under climate change scenarios in China
Source: Nat Commun. 2021 Feb 15;12:1039. doi: 10.1038/s41467-021-21305-1 (PMC7884743; doi:10.1038/s41467-021-21305-1)
Supplement: Supplementary file 1 — Supplementary Information [file 41467_2021_21305_MOESM1_ESM.docx]

**Supplementary materials**

Projecting heat-related excess mortality under climate change scenarios in China

Jun Yang^1,2,3,†^, Maigeng Zhou^4,†^, Zhoupeng Ren^5,†^, Mengmeng Li^6^, Boguang Wang^1,2,3^, De Li Liu^7,8^, Chun-Quan Ou^9^, Peng Yin^4^, Jimin Sun^10^, Shilu Tong^11,12,13^, Hao Wang^1,2,3^, Chunlin Zhang^1,2,3^, Jinfeng Wang^5^, Yuming Guo^14^, Qiyong Liu^10^

^1^ Institute for Environmental and Climate Research, Jinan University, Guangzhou, 511443, China

^2^ Guangdong-Hongkong-Macau Joint Laboratory of Collaborative Innovation for Environmental Quality, Guangzhou, 511443, China

^3^ JNU-QUT Joint Laboratory for Air Quality Science and Management, Jinan University, Guangzhou, 511443, China

^4^ National Center for Chronic and Noncommunicable Disease Control and Prevention, Beijing 100050, China

^5^ State Key Laboratory of Resources and Environmental Information System (LREIS), Institute of Geographic Sciences and Nature Resources Research, Chinese Academy of Sciences, Beijing 100101, China

^6^ State Key Laboratory of Oncology in Southern China, Department of Epidemiology, Cancer Prevention Center, Sun Yat-sen University Cancer Center, Guangzhou 510060, China

^7^ NSW Department of Primary Industries, Wagga Wagga Agricultural Institute, NSW 2650, Australia

^8^ Climate Change Research Centre, University of New South Wales, Sydney, NSW 2052, Australia

^9^ State Key Laboratory of Organ Failure Research, Department of Biostatistics, Guangdong Provincial Key Laboratory of Tropical Disease Research, School of Public Health, Southern Medical University, Guangzhou 510515, China

^10^ State Key Laboratory of Infectious Disease Prevention and Control, Collaborative Innovation Center for Diagnosis and Treatment of Infectious Diseases, National Institute for Communicable Disease Control and Prevention, Chinese Center for Disease Control and Prevention, Beijing 102206, China

^11^ Shanghai Children’s Medical Center, Shanghai Jiao Tong University, Shanghai 200127, China

^12^ School of Public Health and Institute of Environment and Population Health, Anhui Medical University, Hefei, China

^13^ School of Public Health and Institute of Health and Biomedical Innovation, Queensland University of Technology, Brisbane, Australia.

^14^ Department of Epidemiology and Preventive Medicine, School of Public Health and Preventive Medicine, Monash University, Melbourne, Australia

^†^ Co-first authors.

Correspondence to: Jun Yang (yangjun_eci@jnu.edu.cn); Qiyong Liu (liuqiyong@icdc.cn).

**Supplementary Table 1** The information of disease surveillance points during 2007-2013 in China.

| DSP code | DSP site name | Province or municipalities | Urban/rural | Region | Population | | | GDP per capita | Annual mean temperature(^o^C) |
| --- | --- | --- | --- | --- | --- | --- | --- | --- | --- |
|  |  |  |  |  | 0-74 years | 75+ years | Total |  |  |
| 110101 | Dongcheng District | Beijing | Urban | North | 538484 | 34696 | 573180 | 133 | 11.6 |
| 110112 | Tongzhou District | Beijing | Urban | North | 1151601 | 32655 | 1184256 | 29 | 11.8 |
| 120106 | Hongqiao district | Tianjin | Urban | North | 503288 | 28238 | 531526 | 22 | 12.7 |
| 120225 | Ji County | Tianjin | Rural | North | 753697 | 31092 | 784789 | 32 | 11.8 |
| 130205 | Kaiping District | Hebei | Urban | North | 253846 | 8725 | 262571 | 56 | 11.1 |
| 130227 | Qianxi County | Hebei | Rural | North | 378545 | 11583 | 390128 | 83 | 10.1 |
| 130302 | Haigang District | Hebei | Urban | North | 744179 | 21075 | 765254 | 63 | 9.9 |
| 130427 | Ci County | Hebei | Rural | North | 663665 | 14219 | 677884 | 26 | 13.2 |
| 130481 | Wuan City | Hebei | Rural | North | 803608 | 15392 | 819000 | 55 | 13.1 |
| 130702 | Zhangjiakou City | Hebei | Urban | North | 395628 | 14488 | 410116 | 26 | 7.7 |
| 130721 | Xuanhua County | Hebei | Rural | North | 263150 | 10356 | 273506 | 19 | 8.5 |
| 130826 | Fengning Manchu Zizhi County | Hebei | Rural | North | 344466 | 12563 | 357029 | 19 | 6.5 |
| 140107 | Xinghualing District | Shanxi | Urban | North | 614899 | 28685 | 643584 | 52 | 9.5 |
| 140321 | Pingding County | Shanxi | Rural | North | 326799 | 8466 | 335265 | 15 | 11.6 |
| 140427 | Huguan County | Shanxi | Rural | North | 284758 | 6851 | 291609 | 9 | 11.2 |
| 140602 | Shuocheng District | Shanxi | Urban | North | 493399 | 11895 | 505294 | 48 | 8.2 |
| 140826 | Jiang County | Shanxi | Rural | North | 273999 | 7644 | 281643 | 14 | 12.9 |
| 141124 | Lin County | Shanxi | Rural | North | 564322 | 14755 | 579077 | 5 | 9.5 |
| 150103 | Huimin District | Inner Mongolia | Urban | North | 384707 | 9848 | 394555 | 68 | 5.8 |
| 150423 | Bairin Youqi | Inner Mongolia | Rural | North | 172300 | 3243 | 175543 | 24 | 5.7 |
| 150523 | Kailu County | Inner Mongolia | Rural | North | 388818 | 7348 | 396166 | 39 | 7 |
| 150802 | Linhe District | Inner Mongolia | Urban | North | 530180 | 11541 | 541721 | 38 | 8.2 |
| 152524 | Sonid Youqi | Inner Mongolia | Rural | North | 69263 | 1800 | 71063 | 56 | 4.5 |
| 210113 | Shenbei New District | Liaoning | Urban | Northeast | 412040 | 11283 | 423323 | 88 | 7.6 |
| 210204 | Shahekou District | Liaoning | Urban | Northeast | 656015 | 37125 | 693140 | 39 | 10.6 |
| 210311 | Qianshan District | Liaoning | Urban | Northeast | 265471 | 9976 | 275447 | 76 | 9.8 |
| 210682 | Fengcheng City | Liaoning | Rural | Northeast | 522524 | 21409 | 543933 | 50 | 8 |
| 210921 | Fuxin Mongolia Zizhi County | Liaoning | Rural | Northeast | 645438 | 19533 | 664971 | 18 | 8.1 |
| 211021 | Liaoyang County | Liaoning | Rural | Northeast | 554233 | 19276 | 573509 | 33 | 8.9 |
| 220102 | Nanguan District | Jilin | Urban | Northeast | 851363 | 25596 | 876959 | 17 | 5.7 |
| 220183 | Dehui City | Jilin | Rural | Northeast | 728743 | 19626 | 748369 | 34 | 5 |
| 220211 | Fengman District | Jilin | Urban | Northeast | 287673 | 9149 | 296822 | 47 | 4.8 |
| 220582 | Jian City | Jilin | Rural | Northeast | 224601 | 7677 | 232278 | 29 | 6.4 |
| 222405 | Longjing City | Jilin | Rural | Northeast | 169443 | 7791 | 177234 | 14 | 5 |
| 230103 | Nangang District | Heilongjiang | Urban | Northeast | 1300388 | 43469 | 1343857 | 59 | 5 |
| 230208 | Meiris Daur District | Heilongjiang | Urban | Northeast | 162125 | 3727 | 165852 | 12 | 3.6 |
| 230223 | Yian County | Heilongjiang | Rural | Northeast | 468959 | 11076 | 480035 | 10 | 2.8 |
| 230305 | Lishu District | Heilongjiang | Urban | Northeast | 72763 | 3598 | 76361 | 4 | 4.2 |
| 230523 | Baoqing County | Heilongjiang | Rural | Northeast | 388708 | 11779 | 400487 | 25 | 4.2 |
| 230606 | Datong Distrcit | Heilongjiang | Urban | Northeast | 229735 | 4822 | 234557 | 28 | 4.6 |
| 230826 | Huachuan County | Heilongjiang | Rural | Northeast | 198523 | 4304 | 202827 | 10 | 3.4 |
| 310101 | Huangpu District | Shanghai | Urban | East | 396463 | 33428 | 429891 | 183 | 17 |
| 310117 | Songjiang District | Shanghai | Urban | East | 1540913 | 41485 | 1582398 | 57 | 17.2 |
| 320111 | Pukou District | Jiangsu | Urban | East | 687550 | 22748 | 710298 | 61 | 16.3 |
| 320303 | Yunlong District | Jiangsu | Urban | East | 332022 | 13371 | 345393 | 45 | 15.1 |
| 320506 | Wuzhong District | Jiangsu | Urban | East | 1125467 | 31653 | 1157120 | 61 | 17 |
| 320582 | Zhangjiagang City | Jiangsu | Rural | East | 1198575 | 48187 | 1246762 | 149 | 16.1 |
| 320831 | Jinhu County | Jiangsu | Rural | East | 305339 | 15912 | 321251 | 38 | 15.3 |
| 320921 | Xiangshui County | Jiangsu | Rural | East | 491506 | 18374 | 509880 | 32 | 14.4 |
| 330103 | Xiacheng District | Zhejiang | Urban | East | 500798 | 25298 | 526096 | 88 | 17.3 |
| 330283 | Fenghua City | Zhejiang | Rural | East | 467993 | 23704 | 491697 | 46 | 17.6 |
| 330483 | Tongxiang City | Zhejiang | Rural | East | 781153 | 34695 | 815848 | 50 | 17.1 |
| 330523 | Anji County | Zhejiang | Rural | East | 446330 | 20222 | 466552 | 41 | 16.8 |
| 330702 | Wucheng District | Zhejiang | Urban | East | 733549 | 28113 | 761662 | 40 | 18.2 |
| 331123 | Suichang County | Zhejiang | Rural | East | 178016 | 12149 | 190165 | 30 | 18.1 |
| 340181 | Chaohu City | Anhui | Urban | East | 3710904 | 162198 | 3873102 | 5 | 16.2 |
| 340504 | Yushan District | Anhui | Urban | East | 298785 | 10887 | 309672 | 34 | 16.4 |
| 340803 | Daguan District | Anhui | Urban | East | 264707 | 10296 | 275003 | 41 | 16.7 |
| 341181 | Tianchang City | Anhui | Rural | East | 576015 | 26825 | 602840 | 24 | 15.7 |
| 341622 | Mengcheng County | Anhui | Rural | East | 1120507 | 41573 | 1162080 | 10 | 15.6 |
| 341823 | Jing County | Anhui | Rural | East | 285442 | 14113 | 299555 | 16 | 15.8 |
| 350402 | Meilie District | Fujian | Urban | East | 171569 | 4970 | 176539 | 97 | 18.6 |
| 350521 | Huian County | Fujian | Rural | East | 912938 | 31293 | 944231 | 42 | 19.9 |
| 350783 | Jianou City | Fujian | Rural | East | 435863 | 16311 | 452174 | 23 | 18.3 |
| 350822 | Yongaing County | Fujian | Rural | East | 347224 | 15434 | 362658 | 31 | 20.5 |
| 350902 | Jiaocheng District | Fujian | Urban | East | 413900 | 15360 | 429260 | 30 | 18.5 |
| 360102 | Donghu District | Jiangxi | Urban | Central | 554842 | 20647 | 575489 | 51 | 17.5 |
| 360423 | Wuning County | Jiangxi | Rural | Central | 348294 | 11975 | 360269 | 14 | 17.2 |
| 360702 | Zhanggong District | Jiangxi | Urban | Central | 625264 | 17389 | 642653 | 22 | 19.2 |
| 360727 | Longnan County | Jiangxi | Rural | Central | 291058 | 9243 | 300301 | 20 | 20 |
| 360923 | Shanggao County | Jiangxi | Rural | Central | 315536 | 11161 | 326697 | 22 | 18 |
| 370203 | Shibei District | Shandong | Urban | East | 528290 | 29928 | 558218 | 56 | 13 |
| 370213 | Licang District | Shandong | Urban | East | 494390 | 17999 | 512389 | 47 | 12.9 |
| 370323 | Yiyuan County | Shandong | Rural | East | 927534 | 21957 | 949491 | 20 | 12.9 |
| 370403 | Xuecheng District | Shandong | Urban | East | 464325 | 17125 | 481450 | 25 | 14.1 |
| 370602 | Zhifu District | Shandong | Urban | East | 804782 | 25272 | 830054 | 32 | 12.5 |
| 370684 | Penglai City | Shandong | Rural | East | 429851 | 21258 | 451109 | 91 | 12.5 |
| 370785 | Gaomi City | Shandong | Rural | East | 854440 | 41142 | 895582 | 37 | 12.9 |
| 371202 | Laicheng District | Shandong | Urban | East | 947809 | 41726 | 989535 | 34 | 12.8 |
| 371327 | Junan County | Shandong | Rural | East | 841803 | 44573 | 886376 | 20 | 13.5 |
| 410102 | Zhongyuan District | Henan | Urban | Central | 881938 | 23553 | 905491 | 26 | 14.6 |
| 410306 | Jilu District | Henan | Urban | Central | 67040 | 2036 | 69076 | 43 | 12.5 |
| 410323 | Xinan County | Henan | Rural | Central | 456697 | 13501 | 470198 | 55 | 13.6 |
| 410526 | Hua County | Henan | Rural | Central | 1223672 | 39617 | 1263289 | 11 | 14 |
| 410782 | Hui County | Henan | Rural | Central | 719235 | 21200 | 740435 | 28 | 13.6 |
| 411328 | Tanghe County | Henan | Rural | Central | 1242325 | 39937 | 1282262 | 14 | 15.8 |
| 411422 | Sui County | Henan | Rural | Central | 685322 | 25814 | 711136 | 13 | 14.8 |
| 411502 | Shihe District | Henan | Urban | Central | 577469 | 16922 | 594391 | 27 | 16 |
| 420102 | Jiangan District | Hubei | Urban | Central | 859849 | 36108 | 895957 | 59 | 17.2 |
| 420202 | Huangshigang District | Hubei | Urban | Central | 217583 | 6731 | 224314 | 38 | 17.3 |
| 420503 | Wujiagang District | Hubei | Urban | Central | 207293 | 6901 | 214194 | 44 | 16.8 |
| 420625 | Gucheng County | Hubei | Rural | Central | 505845 | 17762 | 523607 | 22 | 16.1 |
| 420923 | Yunmeng County | Hubei | Rural | Central | 509655 | 15144 | 524799 | 21 | 16.7 |
| 429006 | Tianmen City | Hubei | Rural | Central | 1374018 | 44895 | 1418913 | 15 | 17.1 |
| 430103 | Tianxin District | Hunan | Urban | Central | 460972 | 14249 | 475221 | 84 | 17.8 |
| 430181 | Liuyang City | Hunan | Rural | Central | 1226265 | 53204 | 1279469 | 44 | 17.7 |
| 430626 | Pingjiang County | Hunan | Rural | Central | 913767 | 33807 | 947574 | 12 | 17.6 |
| 430702 | Wuling District | Hunan | Urban | Central | 602487 | 18093 | 620580 | 86 | 17.7 |
| 431003 | Suxian District | Hunan | Urban | Central | 389831 | 12919 | 402750 | 36 | 18.1 |
| 431281 | Hongjiang City | Hunan | Rural | Central | 456364 | 21632 | 477996 | 10 | 17.2 |
| 433123 | Fenghuang County | Hunan | Rural | Central | 334684 | 12130 | 346814 | 10 | 17 |
| 440104 | Yuexiu District | Guangdong | Urban | South | 1095966 | 61700 | 1157666 | 138 | 22.2 |
| 440282 | Nanxiong City | Guangdong | Rural | South | 300469 | 15710 | 316179 | 19 | 19.8 |
| 441284 | Sihui City | Guangdong | Rural | South | 523564 | 19309 | 542873 | 45 | 21.8 |
| 441424 | Wuhua County | Guangdong | Rural | South | 1012196 | 38332 | 1050528 | 7 | 21.4 |
| 441502 | Shantou City | Guangdong | Urban | South | 478552 | 13710 | 492262 | 24 | 22 |
| 445302 | Yunfu City Yuncheng District | Guangdong | Urban | South | 307692 | 10453 | 318145 | 17 | 22.4 |
| 450126 | Binyang County | Guangxi | Rural | South | 753006 | 29249 | 782255 | 14 | 21.5 |
| 450205 | Liubei District | Guangxi | Urban | South | 414678 | 13365 | 428043 | 87 | 20.6 |
| 450302 | Xiufeng District | Guangxi | Urban | South | 151390 | 5114 | 156504 | 38 | 19.1 |
| 450521 | Hepu County | Guangxi | Rural | South | 832745 | 38462 | 871207 | 16 | 22.6 |
| 451027 | Lingyun County | Guangxi | Rural | South | 180010 | 5859 | 185869 | 8 | 19.7 |
| 451225 | Luocheng Mulam County | Guangxi | Rural | South | 285432 | 12829 | 298261 | 10 | 19.4 |
| 460108 | Meilan District | Hainan | Urban | South | 339382 | 10159 | 349541 | 45 | 24 |
| 469021 | Dingan County | Hainan | Rural | South | 271589 | 13025 | 284614 | 14 | 24.3 |
| 500101 | Wanzhou District | Chongqing | Urban | Southwest | 1499313 | 63737 | 1563050 | 32 | 17.1 |
| 500111 | Dazu District | Chongqing | Rural | Southwest | 640055 | 31188 | 671243 | 22 | 18.1 |
| 510105 | Qingyang District | Sichuan | Urban | Southwest | 794712 | 33428 | 828140 | 58 | 15.9 |
| 510182 | Pengzhou City | Sichuan | Rural | Southwest | 731801 | 31086 | 762887 | 20 | 15.6 |
| 510411 | Renhe District | Sichuan | Urban | Southwest | 253659 | 6635 | 260294 | 38 | 18.3 |
| 511025 | Zizhong County | Sichuan | Rural | Southwest | 1140218 | 51842 | 1192060 | 11 | 17.3 |
| 511325 | Xichong County | Sichuan | Rural | Southwest | 487662 | 26084 | 513746 | 10 | 17.3 |
| 511823 | Hanyuan County | Sichuan | Rural | Southwest | 312340 | 12068 | 324408 | 10 | 12.5 |
| 513321 | Kangding County | Sichuan | Rural | Southwest | 127915 | 2227 | 130142 | 25 | 9.6 |
| 513434 | Yuexi County | Sichuan | Rural | Southwest | 266022 | 3874 | 269896 | 9 | 13 |
| 520302 | Honghuagang District | Guizhou | Urban | Southwest | 640852 | 15740 | 656592 | 25 | 15.6 |
| 520328 | Meitan County | Guizhou | Rural | Southwest | 365272 | 12086 | 377358 | 8 | 16 |
| 522223 | Yuping Dong Zizhi County | Guizhou | Rural | Southwest | 113897 | 4628 | 118525 | 21 | 17.2 |
| 522623 | Shibing County | Guizhou | Rural | Southwest | 126247 | 4217 | 130464 | 10 | 16.2 |
| 522726 | Dushan County | Guizhou | Rural | Southwest | 255259 | 9953 | 265212 | 9 | 16.6 |
| 530402 | Hongta District | Yunnan | Urban | Southwest | 479098 | 16031 | 495129 | 87 | 15.2 |
| 530423 | Tonghai County | Yunnan | Rural | Southwest | 290446 | 10354 | 300800 | 16 | 16.3 |
| 532627 | Guangnan County | Yunnan | Rural | Southwest | 769324 | 18125 | 787449 | 5 | 17.6 |
| 532823 | Mengla County | Yunnan | Rural | Southwest | 276602 | 5128 | 281730 | 14 | 20.8 |
| 532923 | Xingyun County | Yunnan | Rural | Southwest | 441280 | 14325 | 455605 | 14 | 17.7 |
| 533325 | Lanping Bai Pumi Zizhi County | Yunnan | Rural | Southwest | 208027 | 4965 | 212992 | 11 | 13.3 |
| 540102 | Chengguan District | Tibet | Urban | Southwest | 276628 | 2446 | 279074 | 16 | 7.8 |
| 540127 | Mozhu Gongka County | Tibet | Rural | Southwest | 43794 | 880 | 44674 | 23 | 5.1 |
| 542221 | Naidong County | Tibet | Rural | Southwest | 58868 | 747 | 59615 | 40 | 7.8 |
| 542323 | Jiangzi County | Tibet | Rural | Southwest | 62520 | 983 | 63503 | 19 | 5.8 |
| 542623 | Minlin County | Tibet | Rural | Southwest | 22391 | 443 | 22834 | 21 | 7.5 |
| 610202 | Tongchuan City | Shaanxi | Urban | Northwest | 191781 | 8450 | 200231 | 22 | 12.3 |
| 610326 | Wngyi District | Shaanxi | Rural | Northwest | 292801 | 7189 | 299990 | 18 | 12.6 |
| 610582 | Huayin County | Shaanxi | Rural | Northwest | 250580 | 7533 | 258113 | 19 | 12.2 |
| 610629 | Luochuan County | Shaanxi | Rural | Northwest | 215589 | 5095 | 220684 | 58 | 11 |
| 610921 | Hanyin County | Shaanxi | Rural | Northwest | 239130 | 7017 | 246147 | 11 | 14.3 |
| 620423 | Jingtai County | Gansu | Rural | Northwest | 221221 | 4534 | 225755 | 15 | 9.1 |
| 620503 | Maiji District | Gansu | Urban | Northwest | 538989 | 14279 | 553268 | 15 | 10.5 |
| 620702 | Ganzhou District | Gansu | Rural | Northwest | 499596 | 7837 | 507433 | 20 | 7.5 |
| 620982 | Dunhuang City | Gansu | Rural | Northwest | 183188 | 2839 | 186027 | 28 | 8.5 |
| 623021 | Lintan County | Gansu | Rural | Northwest | 134656 | 2345 | 137001 | 6 | 6.7 |
| 630103 | Chengzhong District | Qinghai | Urban | Northwest | 288154 | 8833 | 296987 | 37 | 5.7 |
| 632121 | Pingan County | Qinghai | Rural | Northwest | 100684 | 2291 | 102975 | 25 | 6.2 |
| 632221 | Menyuan Hui Zizhi County | Qinghai | Rural | Northwest | 145732 | 1978 | 147710 | 2 | 4.3 |
| 640104 | Xingqing District | Ningxia | Urban | Northwest | 663278 | 15028 | 678306 | 28 | 9.4 |
| 640502 | Zhongweicheng District | Ningxia | Rural | Northwest | 372456 | 6150 | 378606 | 22 | 10 |
| 650102 | Tianshan District | Xinjiang | Urban | Northwest | 678823 | 17454 | 696277 | 30 | 6.3 |
| 652925 | Xinhe County | Xinjiang | Rural | Northwest | 168583 | 3481 | 172064 | 10 | 10.1 |
| 653125 | Shache Cpunty | Xinjiang | Rural | Northwest | 752324 | 10061 | 762385 | 5 | 11.9 |
| 653221 | Hetian County | Xinjiang | Rural | Northwest | 266719 | 3222 | 269941 | 5 | 11.8 |
| 654025 | Xinyuan County | Xinjiang | Rural | Northwest | 277997 | 4721 | 282718 | 18 | 3.8 |

**Supplementary Table 2** The 28 general climate models of the 5th phase of Coupled Model Inter-comparison Project.

| GCM | Organization |  | Atmos. Lat × lon (^o^C) | Country | Reference |
| --- | --- | --- | --- | --- | --- |
| BCC-CSM1-1 | Beijing Climate Center, China Meteorological Administration |  | 2.8 × 2.8 | China | (Wu et al., 2013) |
| BCC-CSM1-1-M | Beijing Climate Center, China Meteorological Administration |  |  |  | (Liu et al., 2014) |
| BNU-ESM | Beijing Normal University |  |  |  | (Ji et al., 2014) |
| CanESM2 | Canadian Centre for Climate Modelling and Analysis |  | 2.8 × 2.8 | Canada | (Arora et al., 2011) |
| CCSM4 | National Center for Atmospheric Research |  | 0.9 × 1.3 | USA | (Meehl et al., 2012) |
| CESM1-CAM5 | Centre Européen de Recherche et Formation Avancée en Calcul Scientifique |  |  |  | (Meehl et al., 2013) |
| CMCC-CM  CMCC-CMs | Euro-Mediterranean Center on Climate Change |  | 0.7 × 0.8 | Italy | (Scoccimarro et al., 2011) |
|  |  |  | 3.7 × 3.7 |  | (Weare, Cagnazzo, Fogli, Manzini, & Navarra, 2012) |
| CSIRO-Mk3-6-0 | Organization in collaboration with Queensland Climate Change Centre of Excellence |  | 1.9 × 1.9 | Australia | (Rotstayn et al., 2012) |
| EC-EARTH | EC-EARTH consortium |  | 1.1 × 1.1 | Europe | (Hazeleger et al., 2012) |
| FIO-ESM | The First Institute of Oceanography, SOA, China |  | 2.8 × 2.8 | China | (Bao, Song, & Qiao, 2020) |
| GFDL-CM3  GFDL-ESM2G  GFDL-ESM2M | NOAA Geophysical Fluid Dynamics Laboratory |  | 2.0 × 2.5 | USA | (Griffies et al., 2011) |
|  |  |  | 2.0 × 2.0 |  | (Dunne et al., 2012) |
|  |  |  | 2.0 × 2.5 |  |  |
| GISS-E2-H-CC  GISS-E2-H-R | NASA Goddard Institute for Space Studies |  | 2.0 × 2.5 |  | (Hansen et al., 2007) |
| HadGEM2-AO | National Institute of Meteorological Research, Korea Meteorological Administration |  | 1.9 × 1.2 | South Korea | (Baek et al., 2013) |
| INMCM4 | Institute for Numerical Mathematics |  | 1.5 × 2.0 | Russia | (Volodin, Dianskii, & Gusev, 2010) |
| IPSL-CM5A-MR | Institute Pierre-Simon Laplace |  | 1.3 × 2.5 | France | (Dufresne et al., 2013) |
| IPSL-CM5B-LR |  |  | 1.9 × 3.8 |  |  |
| MIROC5 | Atmosphere and Ocean Research Institute (The University of Tokyo), National Institute for Environmental Studies, and Japan Agency for Marine-Earth Science and Technology |  | 1.4 × 1.4 | Japan | (M. Watanabe et al., 2010) |
| MIROC-ESM |  |  | 2.8 × 2.8 |  | (S. Watanabe et al., 2011) |
| MIROC-ESM-CHEM |  |  | 2.8 × 2.8 |  | (Leng, Tang, & Rayburg, 2015) |
| MPI-ESM-LR | Max-Planck-Institut für Meteorologie (Max Planck Institute for Meteorology) |  | 1.9 × 1.9 |  | (Giorgetta et al., 2013) |
| MPI-ESM-MR |  |  |  | Germany |  |
| MRI-CGCM3 | Meteorological Research Institute[, Japan Meteorological Agency](https://www.researchgate.net/institution/Meteorological_Research_Institute_Japan_Meteorological_Agency) |  | 1.1 × 1.1 | Japan | (YUKIMOTO et al., 2012) |
| NorESM1-M | Norwegian Climate Centre |  | 1.9 × 2.5 | Norway | (Bentsen et al., 2013) |
| NorESM1-ME |  |  |  |  | (Langehaug, Sandø, Årthun, & Ilıcak, 2019) |

**Supplementary Table 3** Summary descriptive statistics on number of deaths in 161 Chinese districts/counties during 2007-2013. P25, P50 and P75 denotes the 25^th^, 50^th^ and 75^th^ percentiles.

| Variables | Total number of deaths | Minimum | P25 | P50 | P75 | Maximum |
| --- | --- | --- | --- | --- | --- | --- |
| Causes |  |  |  |  |  |  |
| Non-accidental mortality | 2,742,717 | 0 | 3 | 6 | 10 | 142 |
| Cardiovascular mortality | 1,274,558 | 0 | 1 | 2 | 5 | 73 |
| Stroke mortality | 670,017 | 0 | 0 | 1 | 3 | 48 |
| IHD mortality | 431,814 | 0 | 0 | 1 | 2 | 44 |
| Respiratory mortality | 397,738 | 0 | 0 | 1 | 2 | 32 |
| COPD mortality | 302,125 | 0 | 0 | 0 | 1 | 28 |
| Gender |  |  |  |  |  |  |
| Male | 1,592,278 | 0 | 2 | 3 | 6 | 86 |
| Female | 1,150,439 | 0 | 1 | 2 | 4 | 61 |
| Age (years) |  |  |  |  |  |  |
| 0-74 | 1,423,850 | 0 | 1 | 3 | 5 | 86 |
| 75+ | 1,318,847 | 0 | 1 | 2 | 5 | 76 |
| Educational level |  |  |  |  |  |  |
| Illiterate | 1,069,714 | 0 | 1 | 2 | 4 | 52 |
| Primary school or higher | 1,532,143 | 0 | 1 | 3 | 6 | 96 |

**Supplementary Table 4** Average values (and standard deviation) of projected daily mean temperature (^o^C) under the RCP4.5 and RCP8.5 scenarios in the 2010s, 2030s, 2050s and 2090s. Δ denotes the change in the average values of daily mean temperature from baseline period (2010s).

| Variables | RCP4.5 | | | | | | | | | | | | | |  | RCP8.5 | | | | | | | | | | | | | | |
| --- | --- | --- | --- | --- | --- | --- | --- | --- | --- | --- | --- | --- | --- | --- | --- | --- | --- | --- | --- | --- | --- | --- | --- | --- | --- | --- | --- | --- | --- | --- |
|  | 2010s |  |  | 2030s |  |  |  | 2050s |  |  |  | 2090s |  |  |  | 2010s |  |  | 2030s |  |  |  | 2050s |  |  |  | 2090s |  |  |  |
|  | Mean | SD |  | Mean | SD | Δ |  | Mean | SD | Δ |  | Mean | SD | Δ |  | Mean | SD |  | Mean | SD | Δ |  | Mean | SD | Δ |  | Mean | SD | Δ |  |
| North | 11.2 | 11.0 |  | 11.8 | 11.1 | 0.6 |  | 12.3 | 11.1 | 1.1 |  | 12.8 | 11.1 | 1.6 |  | 11.2 | 11.1 |  | 12.0 | 11.0 | 0.8 |  | 13.0 | 11.0 | 1.8 |  | 15.2 | 11.0 | 4.0 |  |
| Northeast | 6.7 | 13.2 |  | 7.3 | 13.2 | 0.6 |  | 7.9 | 13.3 | 1.2 |  | 8.4 | 13.2 | 1.7 |  | 6.7 | 13.3 |  | 7.5 | 13.2 | 0.8 |  | 8.6 | 13.1 | 1.9 |  | 11.3 | 12.8 | 4.6 |  |
| Northwest | 10.9 | 9.9 |  | 11.5 | 9.9 | 0.6 |  | 12.0 | 9.9 | 1.1 |  | 12.5 | 9.9 | 1.6 |  | 10.9 | 9.9 |  | 11.7 | 9.9 | 0.8 |  | 12.7 | 9.9 | 1.8 |  | 14.9 | 9.9 | 4.0 |  |
| East | 16.4 | 8.5 |  | 17.0 | 8.6 | 0.6 |  | 17.5 | 8.7 | 1.1 |  | 18.0 | 8.7 | 1.6 |  | 16.4 | 8.6 |  | 17.2 | 8.6 | 0.8 |  | 18.2 | 8.7 | 1.8 |  | 20.2 | 8.8 | 3.8 |  |
| Central | 17.6 | 8.5 |  | 18.1 | 8.6 | 0.5 |  | 18.6 | 8.6 | 1.0 |  | 19.1 | 8.6 | 1.5 |  | 17.6 | 8.6 |  | 18.3 | 8.6 | 0.7 |  | 19.2 | 8.6 | 1.6 |  | 21.1 | 8.7 | 3.5 |  |
| Southwest | 15.3 | 6.2 |  | 15.8 | 6.2 | 0.5 |  | 16.2 | 6.2 | 0.9 |  | 16.7 | 6.3 | 1.4 |  | 15.3 | 6.2 |  | 15.9 | 6.3 | 0.6 |  | 17.6 | 6.2 | 2.3 |  | 18.5 | 6.3 | 3.2 |  |
| South | 22.7 | 5.7 |  | 23.2 | 5.7 | 0.5 |  | 23.7 | 5.6 | 1.0 |  | 24.1 | 5.6 | 1.4 |  | 22.7 | 5.8 |  | 23.3 | 5.7 | 0.6 |  | 24.2 | 5.6 | 1.5 |  | 26.0 | 5.5 | 3.3 |  |
| National | 14.4 | 10.5 |  | 14.9 | 10.5 | 0.5 |  | 15.5 | 10.5 | 1.1 |  | 15.9 | 10.5 | 1.5 |  | 14.4 | 10.6 |  | 15.1 | 10.5 | 0.7 |  | 16.1 | 10.5 | 1.7 |  | 18.2 | 10.3 | 3.8 |  |

**Supplementary Table 5** Heat-related attributable numbers (95%eCI) of death by region, period and climate change scenario, assuming no adaptation or population changes. A distributed lag non-linear model was used to estimate district/county-specific temperature-mortality association with 14 days of lag adjusted for time trends and day of the week, which were pooled in a multivariate meta-analysis. Then, numbers of deaths attributable to high temperatures, defined as temperatures above the optimum temperature, were calculated by regional and national levels. Monte Carlo simulations generating 1,000 samples were computed to produce the empirical confidence interval.

| Region |  |  | RCP 4.5 |  |  |  |  | RCP8.5 |  |
| --- | --- | --- | --- | --- | --- | --- | --- | --- | --- |
|  | 2010s | 2030s | 2050s | 2090s |  | 2010s | 2030s | 2050s | 2090s |
| Northern | 15268 (-5104, 32885) | 17276 (-3642, 35779) | 19503 (-2099, 38739) | 21738 (-827, 42134) |  | 15599 (-4726, 33180) | 18022 (-3108, 36632) | 22439 (-204, 42716) | 34612 (5984, 64408) |
| Northeast | 3888 (468, 7137) | 5420 (601, 10487) | 6997 (635, 14309) | 8468 (665, 18461) |  | 4201 (475, 7991) | 5898 (632, 11287) | 9065 (815, 19079) | 19607 (1874, 40277) |
| Northwest | 1518(-1614, 4205) | 1995 (-2299, 5671) | 2514 (-3008, 7471) | 3012(-3744, 9284) |  | 1530 (-1660, 4168) | 2161 (-2621, 6264) | 3258 (-4136, 9807) | 6396 (-10038, 19822) |
| Eastern | 19312 (6557, 31506) | 24565 (9447, 39363) | 30612 (12192, 50208) | 36399 (14387, 61896) |  | 20070 (6930, 32448) | 26602 (10761, 42484) | 37616 (15958, 61442) | 67046 (27745, 111330) |
| Central | 22441 (-3496, 46508) | 25981 (-771, 50478) | 29763 (1628, 55587) | 33468 (4023, 61339) |  | 22821 (-3067, 46581) | 27470 (313, 52407) | 34226 (4728, 61920) | 53239 (15058, 89906) |
| Southern | 7116 (-1779, 14071) | 8794 (-2612, 17789) | 10983 (-4433, 23658) | 13044 (-6088, 29460) |  | 7156 (-1754, 14213) | 9567 (-3171, 19367) | 13663 (-6944, 29972) | 24818 (-22267, 56899) |
| Southwest | 6969 (-2654, 15022) | 8573 (-4405, 19079) | 10501(-6379, 24813) | 12422 (-8614, 30599) |  | 6886 (-2611, 14869) | 9245 (-4913, 20581) | 12866 (-9423, 31336) | 23349 (-25346, 60601) |
| National | 76364 (7670, 136841) | 92438 (14670, 161354) | 110689 (20776, 194357) | 128346 (24051, 232185) |  | 78115 (8524, 139184) | 98797 (17751, 171106) | 132923 (25787, 234353) | 228728 (22593, 414619) |

**Supplementary Table 6** Relative change (95%eCI) of heat-related excess mortality in comparison to the period of 2010s, stratified by region, period and climate change scenario, assuming no adaptation or population changes. A distributed lag non-linear model was used to estimate district/county-specific temperature-mortality association with 14 days of lag adjusted for time trends and day of the week, which were pooled in a multivariate meta-analysis. Then, relative change in heat-related attributable fraction of mortality in the 2030s, 2050s and 2090s in comparison to the 2010s, were calculated at the regional and national levels. Monte Carlo simulations generating 1,000 samples were computed to produce the empirical confidence interval (eCI). Heat was defined as temperatures above the optimum temperature.

| Region |  | RCP 4.5 |  |  |  | RCP8.5 |  |
| --- | --- | --- | --- | --- | --- | --- | --- |
|  | 2030s | 2050s | 2090s |  | 2030s | 2050s | 2090s |
| Northern | 0.4 (0.0, 1.0) | 0.8 (-0.1, 1.9) | 1.2 (0.0, 3.0) |  | 0.5 (0.0, 1.1) | 1.3 (0.2, 3.0) | 3.5 (0.5, 8.0) |
| Northeast | 0.3 (-0.1, 1.0) | 0.7 (-0.1, 2.0) | 1.0 (-0.1, 2.9) |  | 0.4 (0.0, 0.9) | 1.1 (0.0, 2.8) | 3.4 (0.3, 7.5) |
| Northwest | 0.2 (-0.2, 0.7) | 0.4 (-0.5, 1.3) | 0.5 (-0.8, 2.0) |  | 0.2 (-0.3, 0.8) | 0.6 (-0.9, 2.2) | 1.7 (-3.0, 5.7) |
| Eastern | 0.5 (0.0, 1.2) | 1.0 (0.2, 2.3) | 1.6 (0.3, 3.5) |  | 0.6 (0.0, 1.4) | 1.6 (0.5, 3.2) | 4.3 (1.4, 8.0) |
| Central | 0.4 (-0.1, 1.0) | 0.9 (0.2, 1.9) | 1.3 (0.2, 3.0) |  | 0.5 (0.1, 1.2) | 1.3 (0.3, 2.9) | 3.6 (0.8, 6.9) |
| Southern | 0.4 (-0.3, 1.4) | 1.0 (-0.8, 3.1) | 1.5 (-1.4, 4.7) |  | 0.6 (-0.5, 1.8) | 1.7 (-1.6, 4.8) | 4.6 (-5.7, 11.8) |
| Southwest | 0.3 (-0.3, 0.9) | 0.6 (-0.7, 1.9) | 0.9 (-1.1, 3.0) |  | 0.4 (-0.4, 1.2) | 1.0 (-1.2, 3.1) | 2.7 (-3.9, 7.9) |
| National | 0.4 (0.0, 1.0) | 0.8 (0.0, 2.0) | 1.2 (0.0, 3.1) |  | 0.5 (0.0, 1.2) | 1.3 (-0.1, 3.0) | 3.6 (-0.5, 7.5) |

**Supplementary Table 7** Effect estimates of heat-related excess mortality (95%eCI) during 2010s, 2030s, 2050s and 2090s, stratified by GDP per capita, assuming no adaptation or population changes. Relative change denotes the heat-related excess mortality during 2030s, 2050s and 2090s in comparison to that in the period of 2010s; low and high GDP per capita was classified using the median of this indicator.

| GDP | Effect (%) | RCP4.5 | | | |  | RCP8.5 | | | |
| --- | --- | --- | --- | --- | --- | --- | --- | --- | --- | --- |
|  |  | 2010 | 2030 | 2050 | 2090 |  | 2010 | 2030 | 2050 | 2090 |
| Low | Excess mortality | 2.0 (0.1, 3.7) | 2.4 (0.2, 4.3) | 2.8 (0.3, 5.0) | 3.2 (0.3, 5.9) |  | 2.1 (0.1, 3.8) | 2.5 (0.2, 4.5) | 3.3 (0.4, 5.9) | 5.4 (-0.2, 10.1) |
|  | Relative change | - | 0.4 (-0.1, 0.9) | 0.8 (-0.1, 1.9) | 1.1 (-0.2, 3.0) |  | - | 0.5 (-0.1, 1.1) | 1.2 (-0.3, 2.9) | 3.3 (-1.3, 7.4) |
| High | Excess mortality | 1.6 (0.2, 2.9) | 2.1 (0.5, 3.5) | 2.5 (0.7, 4.4) | 3.0 (0.8, 5.3) |  | 1.7 (0.3, 3.0) | 2.2 (0.5, 3.8) | 3.1 (0.8, 5.3) | 5.5 (1.1, 9.9) |
|  | Relative change | - | 0.4 (-0.0, 1.0) | 0.9 (0.1, 2.0) | 1.3 (0.1, 3.2) |  | - | 0.5 (0.0, 1.2) | 1.4 (0.1, 3.0) | 3.8 (0.1, 7.7) |

**Supplementary Table 8** Heat-related excess mortality (%) by individual characteristic, period and climate change scenario (95%eCI), assuming no adaptation or population changes. A distributed lag non-linear model was used to estimate the district/county-specific temperature-mortality association with 14 days of lag adjusted for time trends and day of the week, which were pooled in a multivariate meta-analysis. Then, estimates of attributable fraction of deaths due to high temperature, defined as temperatures above the optimum temperature, were calculated by regional and national levels. Monte Carlo simulations generating 1000 samples were computed to produce the empirical confidence intervals. The analyses were separately repeated according to cause of death and individual characteristics.

| Variables | RCP 4.5 | | | |  | RCP8.5 | | | |
| --- | --- | --- | --- | --- | --- | --- | --- | --- | --- |
|  | 2010s | 2030s | 2050s | 2090s |  | 2010s | 2030s | 2050s | 2090s |
| Causes |  |  |  |  |  |  |  |  |  |
| Non-accidental mortality | 1.8 (0.2, 3.3) | 2.2 (0.4, 3.9) | 2.6 (0.5, 4.6) | 3.1 (0.6, 5.5) |  | 1.9 (0.2, 3.3) | 2.4 (0.4, 4.1) | 3.2 (0.6, 5.6) | 5.5 (0.5, 9.9) |
| Cardiovascular mortality | 1.7 (0.8, 2.4) | 2.1 (1.0, 3.2) | 2.7 (1.2, 4.2) | 3.2 (1.3, 5.3) |  | 1.7 (0.8, 2.5) | 2.3 (1.1, 3.5) | 3.4 (1.4, 5.3) | 6.3 (2.3, 10.2) |
| Stroke mortality | 1.7 (0.3, 2.9) | 2.2 (0.4, 3.7) | 2.7 (0.6, 4.7) | 3.2 (0.6, 5.9) |  | 1.8 (0.3, 3.0) | 2.3 (0.5, 4.0) | 3.3 (0.7, 5.9) | 6.1 (0.7, 11.0) |
| IHD mortality | 1.5 (0.3, 2.6) | 2.0 (0.4, 3.4) | 2.4 (0.5, 4.4) | 2.9 (0.5, 5.5) |  | 1.6 (0.3, 2.7) | 2.1 (0.5, 3.7) | 3.0 (0.5, 5.5) | 5.7 (0.5, 10.4) |
| Respiratory mortality | 2.8 (-1.5, 6.2) | 3.3 (-1.5, 7.1) | 3.9 (-1.4, 8.3) | 4.5 (-1.3, 9.4) |  | 2.8 (-1.5, 6.3) | 3.5 (-1.4, 7.5) | 4.7 (-1.3, 9.5) | 7.7 (-1.6, 14.7) |
| COPD mortality | 1.9 (-1.0, 4.0) | 2.3 (-0.9, 4.6) | 2.7 (-0.9, 5.4) | 3.1 (-1.0, 6.3) |  | 1.9 (-0.9, 4.0) | 2.4 (-0.9, 4.9) | 3.2 (-1.1, 6.4) | 5.5 (-1.7, 10.3) |
| Gender |  |  |  |  |  |  |  |  |  |
| Male | 1.3 (0.9, 1.7) | 1.7 (1.0, 2.3) | 2.1 (1.1, 3.2) | 2.5 (1.1, 4.2) |  | 1.3 (0.9, 1.8) | 1.8 (1.1, 2.6) | 2.6 (1.2, 4.2) | 4.8 (1.3, 8.5) |
| Female | 2.2 (-0.2, 4.2) | 2.6 (0.1, 4.9) | 3.1 (0.3, 5.7) | 3.7 (0.5, 6.6) |  | 2.2 (-0.1, 4.3) | 2.8 (0.2, 5.1) | 3.8 (0.6, 6.6) | 6.5 (1.3, 10.9) |
| Age (years) |  |  |  |  |  |  |  |  |  |
| 0-74 | 1.7 (-0.4, 3.6) | 2.0 (-0.1, 3.9) | 2.3 (0.1, 4.4) | 2.7 (0.3, 4.9) |  | 1.7 (-0.3, 3.6) | 2.1 (-0.0, 4.1) | 2.8 (0.4, 4.9) | 4.6 (0.8, 8.0) |
| 75+ | 1.8 (0.8, 2.8) | 2.3 (1.0, 3.6) | 2.9 (1.2, 4.7) | 3.5 (1.4, 5.9) |  | 1.9 (0.8, 2.9) | 2.5 (1.1, 3.9) | 3.6 (1.5, 5.9) | 6.6 (2.5, 10.8) |
| Educational level |  |  |  |  |  |  |  |  |  |
| Illiterate | 1.9 (-0.1, 3.7) | 2.4 (-0.1, 4.6) | 2.9 (-0.1, 5.7) | 3.5 (-0.1, 6.9) |  | 2.0 (-0.1, 3.7) | 2.6 (-0.1, 4.9) | 3.6 (-0.1, 6.9) | 6.4 (-0.5, 12.1) |
| Primary school or higher | 1.9 (-0.2, 3.8) | 2.3 (0.1, 4.2) | 2.7 (0.4, 4.6) | 3.0 (0.7, 5.2) |  | 1.9 (-0.2, 3.8) | 2.4 (0.2, 4.3) | 3.2 (0.8, 5.3) | 5.3 (2.0, 8.7) |

**Supplementary Table 9** Heat-related attributable numbers of death by individual characteristics, period and climate change scenario (95%eCI), assuming no adaptation or population changes. A distributed lag non-linear model was used to estimate district/county-specific temperature-mortality association with 14 days of lag adjusted for time trends and day of the week, which were pooled in a multivariate meta-analysis. Then, numbers of deaths attributable to high temperature, defined as temperatures above the optimum temperature, were calculated at the national level. Monte Carlo simulations generating 1,000 samples were computed to produce the empirical confidence interval (eCI). The analyses were separately repeated according to cause of death and individual characteristics.

| Variables | RCP 4.5 | | | |  | RCP8.5 | | | |
| --- | --- | --- | --- | --- | --- | --- | --- | --- | --- |
|  | 2010s | 2030s | 2050s | 2090s |  | 2010s | 2030s | 2050s | 2090s |
| Causes |  |  |  |  |  |  |  |  |  |
| Non-accidental mortality | 76364 (7670, 136841) | 92438 (14670, 161354) | 110689 (20776, 194357) | 128346 (24051, 232185) |  | 78115 (8524, 139184) | 98797 (17751, 171106) | 132923 (25787, 234353) | 228728 (22593, 414619) |
| Cardiovascular mortality | 32144 (16043, 46540) | 41531 (19567, 62011) | 52163 (22804, 81503) | 62596 (25419, 103891) |  | 33051 (16077, 48031) | 45172 (21151, 68030) | 65200 (27470, 103971) | 122006 (45041, 197769) |
| Stroke mortality | 17487 (2883, 29698) | 22141 (4515, 37608) | 27406 (5923, 48007) | 32594 (6583, 59826) |  | 17911 (2918, 30427) | 23946 (5433, 40787) | 33923 (7132, 60128) | 62437 (7431, 111847) |
| IHD mortality | 9980 (1682, 17099) | 12760 (2701, 22041) | 15907 (3151, 28546) | 19002 (3357, 35626) |  | 10287 (1725, 17707) | 13837 (2983, 23975) | 19793 (3506, 35797) | 36975 (3559, 67794) |
| Respiratory mortality | 16955 (-9215, 37934) | 20283 (-8899, 43439) | 23981 (-8395, 50291) | 27558 (-7986, 57436) |  | 17186 (-9201, 38227) | 21572 (-8829, 45554) | 28529 (-7954, 58106) | 47168 (-9968, 89509) |
| COPD mortality | 8795 (-4473, 18649) | 10558 (-4263, 21559) | 12529 (-4235, 25233) | 14471 (-4801, 29320) |  | 8913 (-4399, 18757) | 11246 (-4185, 22802) | 14974 (-4959, 29670) | 25322 (-7772, 48003) |
| Gender |  |  |  |  |  |  |  |  |  |
| Male | 31742 (22274, 41880) | 40474 (25181, 56746) | 50448 (26860, 77999) | 60238 (27794, 101729) |  | 32516 (22134, 42967) | 43849 (26058, 63551) | 62847 (28790, 102183) | 117400 (31336, 206880) |
| Female | 38045 (-3306, 74674) | 46215 (1965, 85448) | 55377 (6048, 99682) | 64298 (9012, 115859) |  | 38766 (-2623, 75589) | 49362 (3761, 89632) | 66533 (10409, 116830) | 113965 (23243, 191610) |
| Age (years) |  |  |  |  |  |  |  |  |  |
| 0-74 | 36987 (-8183, 78521) | 43435 (-2996, 85686) | 50709 (1614, 94491) | 57789 (6135, 106147) |  | 37486 (-7528, 78828) | 45855 (-1031, 88417) | 59728 (8130, 107142) | 99240 (17503, 172893) |
| 75+ | 37210 (16674, 56413) | 47401 (20943, 72954) | 59043 (25047, 94532) | 70396 (28018, 118271) |  | 38183 (16809, 58116) | 51426 (22949, 79515) | 73202 (30471, 118428) | 133711 (50653, 218508) |
| Educational level |  |  |  |  |  |  |  |  |  |
| Illiterate | 31373 (-1648, 60354) | 39359 (-1564, 74806) | 48251 (-1733, 93177) | 56930 (-1902, 113115) |  | 32100 (-1545, 61521) | 42429 (-1377, 80129) | 59021 (-2271, 113402) | 104547 (-7945, 199641) |
| Primary school or higher | 44703 (-5354, 88804) | 52709 (2276, 97366) | 61964 (10215, 108231) | 70966.0 (15911, 122163) |  | 45384 (-4517, 89355) | 55842 (5334, 100226) | 73433 (19383, 123010) | 123729 (46916, 201895) |

**Supplementary Table 10** Relative change (% and 95%eCI) of heat-related excess mortality in comparison to the period of 2010s, stratified by individual characteristics, assuming no adaptation or population changes. A distributed lag non-linear model was used to estimate district/county-specific temperature-mortality association with 14 days of lag adjusted for time trends and day of the week, which were pooled in a multivariate meta-analysis. Then, relative change in heat-related attributable fraction of mortality in the 2030s, 2050s and 2090s in comparison to the 2010s, were calculated by individual characteristics. Monte Carlo simulations generating 1,000 samples were computed to produce the empirical confidence interval (eCI). Heat was defined as temperatures above the optimum temperature.

| Variables |  | RCP 4.5 |  |  |  |  | RCP8.5 |  |  |
| --- | --- | --- | --- | --- | --- | --- | --- | --- | --- |
|  | 2030s | 2050s | 2070s | 2090s |  | 2030s | 2050s | 2070s | 2090s |
| Causes |  |  |  |  |  |  |  |  |  |
| Non-accidental mortality | 0.4 (0.0, 1.0) | 0.8 (0.0, 2.0) | 1.1 (0.0, 2.9) | 1.2 (0.0, 3.1) |  | 0.5 (0.0, 1.2) | 1.3 (-0.1, 3.0) | 2.3 (-0.2, 5.0) | 3.6 (-0.5, 7.5) |
| Cardiovascular mortality | 0.5 (0.0, 1.1) | 1.0 (0.2, 2.1) | 1.4 (0.3, 3.1) | 1.6 (0.3, 3.4) |  | 0.6 (0.1, 1.3) | 1.7 (0.5, 3.2) | 3.0 (0.8, 5.4) | 4.6 (1.3, 8.0) |
| Stroke mortality | 0.5 (0.0, 1.2) | 1.0 (0.0, 2.3) | 1.3 (0.0, 3.3) | 1.5 (0.0, 3.6) |  | 0.6 (0.0, 1.3) | 1.6 (0.0, 3.4) | 2.8 (0.0, 5.7) | 4.4 (-0.2, 8.6) |
| IHD mortality | 0.4 (0.0, 1.1) | 0.9 (0.0, 2.2) | 1.2 (0.0, 3.2) | 1.4 (0.0, 3.4) |  | 0.5 (0.0, 1.3) | 1.5 (0.0, 3.3) | 2.6 (-0.1, 5.5) | 4.1 (-0.3, 8.3) |
| Respiratory mortality | 0.5 (-0.1, 1.4) | 1.2 (-0.2, 2.7) | 1.6 (-0.3, 3.8) | 1.7 (-0.3, 4.2) |  | 0.7 (-0.1, 1.6) | 1.9 (-0.3, 4.1) | 3.2 (-0.7, 6.6) | 4.9 (-1.2, 9.6) |
| COPD mortality | 0.4 (-0.1, 1.0) | 0.8 (-0.2, 1.9) | 1.1 (-0.3, 2.8) | 1.2 (-0.4, 3.0) |  | 0.5 (-0.2, 1.2) | 1.3 (-0.4, 3.0) | 2.3 (-0.9, 4.9) | 3.5 (-1.5, 7.2) |
| Gender |  |  |  |  |  |  |  |  |  |
| Male | 0.4 (0.0, 0.9) | 0.8 (0.1, 1.8) | 1.1 (0.1, 2.6) | 1.2 (0.1, 2.8) |  | 0.5 (0.1, 1.0) | 1.2 (0.2, 2.7) | 2.2 (0.2, 4.6) | 3.5 (0.3, 7.0) |
| Female | 0.5 (0.0, 1.1) | 1.0 (0.1, 2.2) | 1.3 (0.1, 3.1) | 1.5 (0.1, 3.4) |  | 0.6 (0.1, 1.3) | 1.6 (0.2, 3.2) | 2.8 (0.3, 5.4) | 4.3 (0.4, 7.9) |
| Age (years) |  |  |  |  |  |  |  |  |  |
| 0-74 | 0.3 (0.0, 0.7) | 0.6 (0.1, 1.5) | 0.9 (0.1, 2.2) | 1.0 (0.1, 2.3) |  | 0.4 (0.0, 0.9) | 1.0 (0.1, 2.2) | 1.8 (0.0, 3.9) | 2.8 (-0.2, 5.9) |
| 75+ | 0.5 (0.0, 1.1) | 1.1 (0.3, 2.3) | 1.5 (0.3, 3.3) | 1.6 (0.3, 3.5) |  | 0.7 (0.1, 1.4) | 1.7 (0.5, 3.4) | 3.1 (0.9, 5.6) | 4.7 (1.5, 8.4) |
| Educational level |  |  |  |  |  |  |  |  |  |
| Illiterate | 0.5 (-0.0, 1.3) | 1.0 (-0.1, 2.5) | 1.4 (-0.2, 3.6) | 1.6 (-0.2, 3.9) |  | 0.6 (-0.1, 1.5) | 1.6 (-0.2, 3.7) | 2.9 (-0.5, 6.1) | 4.4 (-0.9, 9.1) |
| Primary school or higher | 0.3 (0.0, 0.8) | 0.7 (0.2, 1.6) | 1.0 (0.2, 2.3) | 1.1 (0.2, 2.5) |  | 0.4 (0.1, 0.9) | 1.2 (0.4, 2.4) | 2.2 (0.6, 4.2) | 3.4 (0.8, 6.4) |

**Supplementary Table 11** The heat-related attributable number of deaths for different age groups under six population scenarios (no change, SSP1, SSP2, SSP3, SSP4 and SSP5) under RCP4.5 by region in China.

|  |  |  |  | 0-74 years | |  |  |  |  | 75+ years | | |  |  |  |  |  | Total |  |  |  |
| --- | --- | --- | --- | --- | --- | --- | --- | --- | --- | --- | --- | --- | --- | --- | --- | --- | --- | --- | --- | --- | --- |
| Decades |  | No change | SSP1 | SSP2 | SSP3 | SSP4 | SSP5 |  | No change | SSP1 | SSP2 | SSP3 | SSP4 | SSP5 |  | No change | SSP1 | SSP2 | SSP3 | SSP4 | SSP5 |
|  | North | 10630 | 10941 | 10887 | 10811 | 10922 | 10950 |  | 7266 | 21375 | 19742 | 18391 | 20167 | 21121 |  | 17896 | 32316 | 30629 | 29202 | 31089 | 32071 |
|  | Northeast | 2711 | 2668 | 2669 | 2669 | 2666 | 2666 |  | 4063 | 15362 | 14469 | 13517 | 14621 | 15427 |  | 6774 | 18030 | 17138 | 16186 | 17287 | 18093 |
|  | Northwest | 1290 | 1238 | 1268 | 1294 | 1234 | 1239 |  | 735 | 2214 | 2106 | 1988 | 2093 | 2218 |  | 2025 | 3452 | 3374 | 3282 | 3327 | 3457 |
| 2030s | East | 10074 | 9904 | 10013 | 10102 | 9887 | 9907 |  | 15670 | 38655 | 36348 | 34200 | 36552 | 38363 |  | 25744 | 48559 | 46361 | 44302 | 46439 | 48270 |
|  | Central | 19164 | 18755 | 19003 | 19253 | 18713 | 18709 |  | 8532 | 23434 | 22006 | 20133 | 22148 | 23939 |  | 27696 | 42189 | 41009 | 39386 | 40861 | 42648 |
|  | South | 4134 | 4217 | 4237 | 4248 | 4203 | 4218 |  | 6838 | 14251 | 13301 | 12492 | 13486 | 14120 |  | 10972 | 18468 | 17538 | 16740 | 17689 | 18338 |
|  | Southwest | 3618 | 3287 | 3417 | 3543 | 3280 | 3281 |  | 6937 | 18413 | 17809 | 16840 | 17465 | 18720 |  | 10555 | 21700 | 21226 | 20383 | 20745 | 22001 |
|  | North | 11581 | 9696 | 10017 | 10217 | 9691 | 9772 |  | 8518 | 63379 | 52773 | 44848 | 54266 | 61558 |  | 20099 | 73075 | 62790 | 55065 | 63957 | 71330 |
|  | Northeast | 3361 | 2355 | 2481 | 2636 | 2375 | 2346 |  | 4980 | 52103 | 45975 | 38555 | 46179 | 52521 |  | 8341 | 54458 | 48456 | 41191 | 48554 | 54867 |
|  | Northwest | 1637 | 1212 | 1308 | 1410 | 1202 | 1212 |  | 876 | 7097 | 6161 | 5223 | 6147 | 7138 |  | 2513 | 8309 | 7469 | 6633 | 7349 | 8350 |
| 2050s | East | 11867 | 9039 | 9598 | 10109 | 9040 | 9083 |  | 19588 | 113114 | 97227 | 84284 | 97770 | 111240 |  | 31455 | 122153 | 106825 | 94393 | 106810 | 120323 |
|  | Central | 21075 | 16130 | 17206 | 18499 | 16088 | 15779 |  | 10429 | 67289 | 56793 | 43974 | 57964 | 72348 |  | 31504 | 83419 | 73999 | 62473 | 74052 | 88127 |
|  | South | 4779 | 4006 | 4171 | 4304 | 3963 | 4033 |  | 8414 | 44864 | 37786 | 32744 | 38914 | 43423 |  | 13193 | 48870 | 41957 | 37048 | 42877 | 47456 |
|  | Southwest | 3968 | 2669 | 2963 | 3318 | 2657 | 2622 |  | 8492 | 48005 | 42895 | 35769 | 41800 | 50820 |  | 12460 | 50674 | 45858 | 39087 | 44457 | 53442 |
|  | North | 12499 | 5630 | 7119 | 9147 | 5414 | 5712 |  | 9815 | 79310 | 56445 | 39665 | 56162 | 77405 |  | 22314 | 84940 | 63564 | 48812 | 61576 | 83117 |
|  | Northeast | 3980 | 1262 | 1771 | 2604 | 1257 | 1248 |  | 5804 | 63836 | 50416 | 34366 | 48601 | 64550 |  | 9784 | 65098 | 52187 | 36970 | 49858 | 65798 |
|  | Northwest | 1972 | 772 | 1018 | 1406 | 728 | 771 |  | 1008 | 8063 | 5686 | 3819 | 5709 | 8135 |  | 2980 | 8835 | 6704 | 5225 | 6437 | 8906 |
| 2090s | East | 13597 | 5577 | 7203 | 9735 | 5329 | 5614 |  | 23291 | 127928 | 95297 | 67113 | 91799 | 126559 |  | 36888 | 133505 | 102500 | 76848 | 97128 | 132173 |
|  | Central | 22882 | 9271 | 12365 | 16790 | 8843 | 8607 |  | 12327 | 79605 | 53211 | 33367 | 56853 | 90274 |  | 35209 | 88876 | 65576 | 50157 | 65696 | 98881 |
|  | South | 5386 | 2454 | 3065 | 3982 | 2304 | 2507 |  | 9904 | 58941 | 42061 | 29304 | 42023 | 56146 |  | 15290 | 61395 | 45126 | 33286 | 44327 | 58653 |
|  | Southwest | 4267 | 1433 | 2012 | 2952 | 1371 | 1317 |  | 10052 | 53815 | 38996 | 26456 | 38879 | 61579 |  | 14319 | 55248 | 41008 | 29408 | 40250 | 62896 |

**Supplementary Table 12** The heat-related attributable number of deaths for different age groups under six population scenarios (no change, SSP1, SSP2, SSP3, SSP4 and SSP5) under RCP8.5 by region in China.

| Decades | | 0-74 years | | | | | |  | 75+ years | | | | | |  | Total | | | | | |
| --- | --- | --- | --- | --- | --- | --- | --- | --- | --- | --- | --- | --- | --- | --- | --- | --- | --- | --- | --- | --- | --- |
|  |  | No change | SSP1 | SSP2 | SSP3 | SSP4 | SSP5 |  | No change | SSP1 | SSP2 | SSP3 | SSP4 | SSP5 |  | No change | SSP1 | SSP2 | SSP3 | SSP4 | SSP5 |
|  | North | 10949 | 11270 | 11214 | 11136 | 11250 | 11279 |  | 7679 | 22592 | 20865 | 19438 | 21315 | 22323 |  | 18628 | 33862 | 32079 | 30574 | 32565 | 33602 |
|  | Northeast | 2920 | 2875 | 2876 | 2875 | 2872 | 2872 |  | 4330 | 16372 | 15420 | 14406 | 15583 | 16441 |  | 7250 | 19247 | 18296 | 17281 | 18455 | 19313 |
|  | Northwest | 1399 | 1343 | 1375 | 1404 | 1339 | 1343 |  | 784 | 2362 | 2247 | 2121 | 2233 | 2366 |  | 2183 | 3705 | 3622 | 3525 | 3572 | 3709 |
| 2030s | East | 10653 | 10473 | 10589 | 10684 | 10455 | 10477 |  | 17018 | 41978 | 39473 | 37140 | 39694 | 41661 |  | 27671 | 52451 | 50062 | 47824 | 50149 | 52138 |
|  | Central | 19920 | 19494 | 19753 | 20011 | 19451 | 19447 |  | 9278 | 25486 | 23932 | 21895 | 24087 | 26034 |  | 29198 | 44980 | 43685 | 41906 | 43538 | 45481 |
|  | South | 4347 | 4435 | 4456 | 4467 | 4420 | 4436 |  | 7396 | 15412 | 14385 | 13510 | 14585 | 15271 |  | 11743 | 19847 | 18841 | 17977 | 19005 | 19707 |
|  | Southwest | 3768 | 3424 | 3559 | 3690 | 3416 | 3417 |  | 7460 | 19800 | 19150 | 18108 | 18780 | 20130 |  | 11228 | 23224 | 22709 | 21798 | 22196 | 23547 |
|  | North | 12803 | 10719 | 11074 | 11295 | 10714 | 10803 |  | 10204 | 75928 | 63222 | 53728 | 65011 | 73746 |  | 23007 | 86647 | 74296 | 65023 | 75725 | 84549 |
|  | Northeast | 4269 | 2991 | 3151 | 3348 | 3017 | 2980 |  | 6100 | 63828 | 56320 | 47231 | 56571 | 64340 |  | 10369 | 66819 | 59471 | 50579 | 59588 | 67320 |
|  | Northwest | 2132 | 1579 | 1704 | 1836 | 1566 | 1578 |  | 1078 | 8736 | 7585 | 6429 | 7567 | 8787 |  | 3210 | 10315 | 9289 | 8265 | 9133 | 10365 |
| 2050s | East | 13962 | 10634 | 11291 | 11893 | 10636 | 10686 |  | 24060 | 138938 | 119425 | 103527 | 120091 | 136636 |  | 38022 | 149572 | 130716 | 115420 | 130727 | 147322 |
|  | Central | 23309 | 17840 | 19030 | 20460 | 17794 | 17452 |  | 12676 | 81790 | 69032 | 53451 | 70456 | 87940 |  | 35985 | 99630 | 88062 | 73911 | 88250 | 105392 |
|  | South | 5573 | 4671 | 4864 | 5019 | 4621 | 4702 |  | 10333 | 55095 | 46403 | 40212 | 47789 | 53326 |  | 15906 | 59766 | 51267 | 45231 | 52410 | 58028 |
|  | Southwest | 4342 | 2920 | 3242 | 3631 | 2907 | 2869 |  | 10404 | 58816 | 52556 | 43825 | 51214 | 62266 |  | 14746 | 61736 | 55798 | 47456 | 54121 | 65135 |
|  | North | 17758 | 7998 | 10114 | 12995 | 7692 | 8114 |  | 17152 | 138595 | 98637 | 69315 | 98144 | 135265 |  | 34910 | 146593 | 108751 | 82310 | 105836 | 143379 |
|  | Northeast | 9122 | 2892 | 4059 | 5967 | 2882 | 2860 |  | 11464 | 126079 | 99575 | 67875 | 95989 | 127489 |  | 20586 | 128971 | 103634 | 73842 | 98871 | 130349 |
|  | Northwest | 4251 | 1665 | 2194 | 3030 | 1569 | 1663 |  | 1937 | 15488 | 10922 | 7336 | 10966 | 15625 |  | 6188 | 17153 | 13116 | 10366 | 12535 | 17288 |
| 2090s | East | 22762 | 9335 | 12057 | 16296 | 8920 | 9398 |  | 42617 | 234079 | 174372 | 122802 | 167971 | 231575 |  | 65379 | 243414 | 186429 | 139098 | 176891 | 240973 |
|  | Central | 32254 | 13068 | 17430 | 23668 | 12465 | 12133 |  | 22526 | 145469 | 97236 | 60974 | 103891 | 164963 |  | 54780 | 158537 | 114666 | 84642 | 116356 | 177096 |
|  | South | 8910 | 4059 | 5071 | 6588 | 3812 | 4146 |  | 18128 | 107876 | 76981 | 53633 | 76911 | 102760 |  | 27038 | 111935 | 82052 | 60221 | 80723 | 106906 |
|  | Southwest | 5759 | 1934 | 2715 | 3984 | 1850 | 1778 |  | 18654 | 99865 | 72365 | 49095 | 72148 | 114273 |  | 24413 | 101799 | 75080 | 53079 | 73998 | 116051 |

**Supplementary Table 13** Sensitivity analyses of projected heat-related excess non-accidental mortality (% and 95% eCI) under the RCP4.5 and RCP8.5 scenarios in 2010s, 2050s and 2090s, assuming no adaptation or population changes. Sensitivity analyses were performed by changing the maximum lag for the temperature-lag-mortality dimensional relationship from 8 to 21 days, the degrees of freedom for long-term and seasonal trend of mortality from 6 to 10 per year. We separately included the daily mean relative humidity and mean wind speed in the main model using a natural cubic spline with 3 degrees of freedom as another sensitivity analyses. And we additionally adjusted for the two-day average concentrations of fine particulate matter and ozone using a natural cubic spline with 3 df using data during 2011-2013.

|  |  | RCP4.5 |  |  |  | RCP8.5 |  |
| --- | --- | --- | --- | --- | --- | --- | --- |
| Model choices | 2010s | 2050s | 2090s |  | 2010s | 2050s | 2090s |
| Lag period: 8 days | 1.5 (-0.1, 2.9) | 2.3 (0.2, 4.3) | 2.7 (0.3, 5.2) |  | 1.5 (-0.1, 3.0) | 2.8 (0.4, 5.2) | 5.1 (1.0, 9.3) |
| Lag period:10 days | 1.4 (0.0, 2.7) | 2.2 (0.3, 4.1) | 2.6 (0.3, 5.1) |  | 1.4 (0.0, 2.7) | 2.7 (0.4, 5.1) | 5.0 (0.6, 9.4) |
| Lag period: 21 days | 1.4 (0.2, 2.5) | 2.3 (0.1, 4.2) | 2.7 (0.1, 5.4) |  | 1.4 (0.2, 2.5) | 2.8 (0.0, 5.4) | 5.2 (-0.4, 10.2) |
| df for per year: 6 | 1.9 (0.5, 3.0) | 2.6 (0.8, 4.1) | 2.9 (1.0, 4.8) |  | 1.9 (0.5, 3.1) | 3.0 (1.1, 4.9) | 5.0 (1.9, 8.2) |
| df for per year: 8 | 1.4 (0.2, 2.6) | 2.2 (0.4, 4.0) | 2.7 (0.4, 5.0) |  | 1.5 (0.2, 2.6) | 2.8 (0.5, 5.0) | 5.1 (0.6, 9.3) |
| df for per year: 10 | 1.5 (0.2, 2.7) | 2.4 (0.3, 4.5) | 2.8 (0.2, 5.5) |  | 1.5 (0.2, 2.8) | 2.9 (0.2, 5.6) | 5.3 (-0.2, 10.2) |
| Relative humidity | 1.8 (0.1, 3.2) | 2.6 (0.5, 4.6) | 3.0 (0.6, 5.5) |  | 1.9 (0.1, 3.4) | 3.2 (0.6, 5.5) | 5.4 (0.7, 9.6) |
| Wind speed | 1.8 (0.2, 3.2) | 2.6 (0.6, 4.5) | 3.1 (0.7, 5.3) |  | 1.9 (0.2, 3.3) | 3.2 (0.8, 5.4) | 5.4 (1.1, 9.3) |
| PM_2.5_+O_3_ | 1.5 (0.4,2.5) | 2.4 (0.6, 4.1) | 2.8 (0.6, 5.2) |  | 1.5 (0.4, 2.6) | 2.9 (0.7, 5.2) | 5.4 (0.9, 9.8) |

**
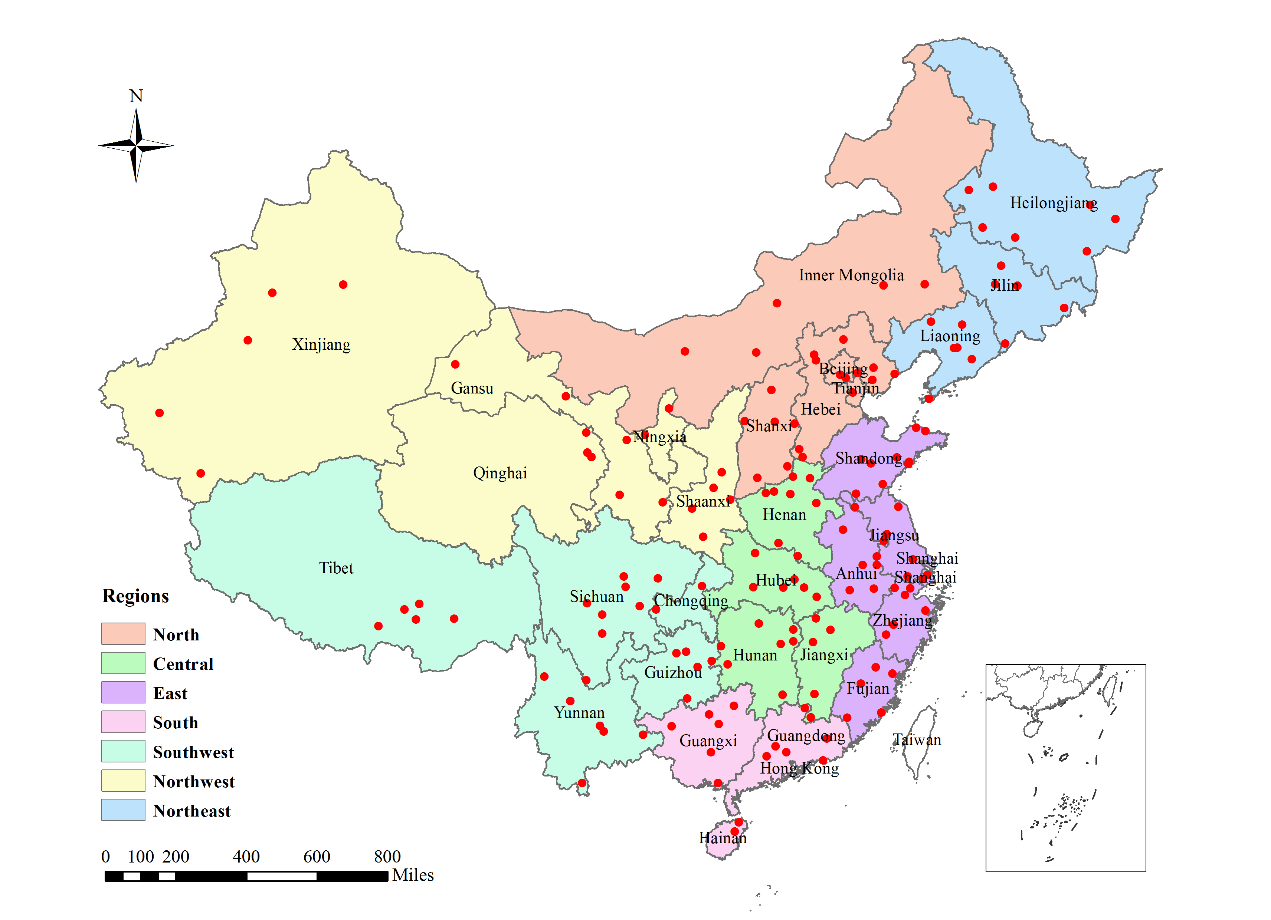
**

**b**

**a**


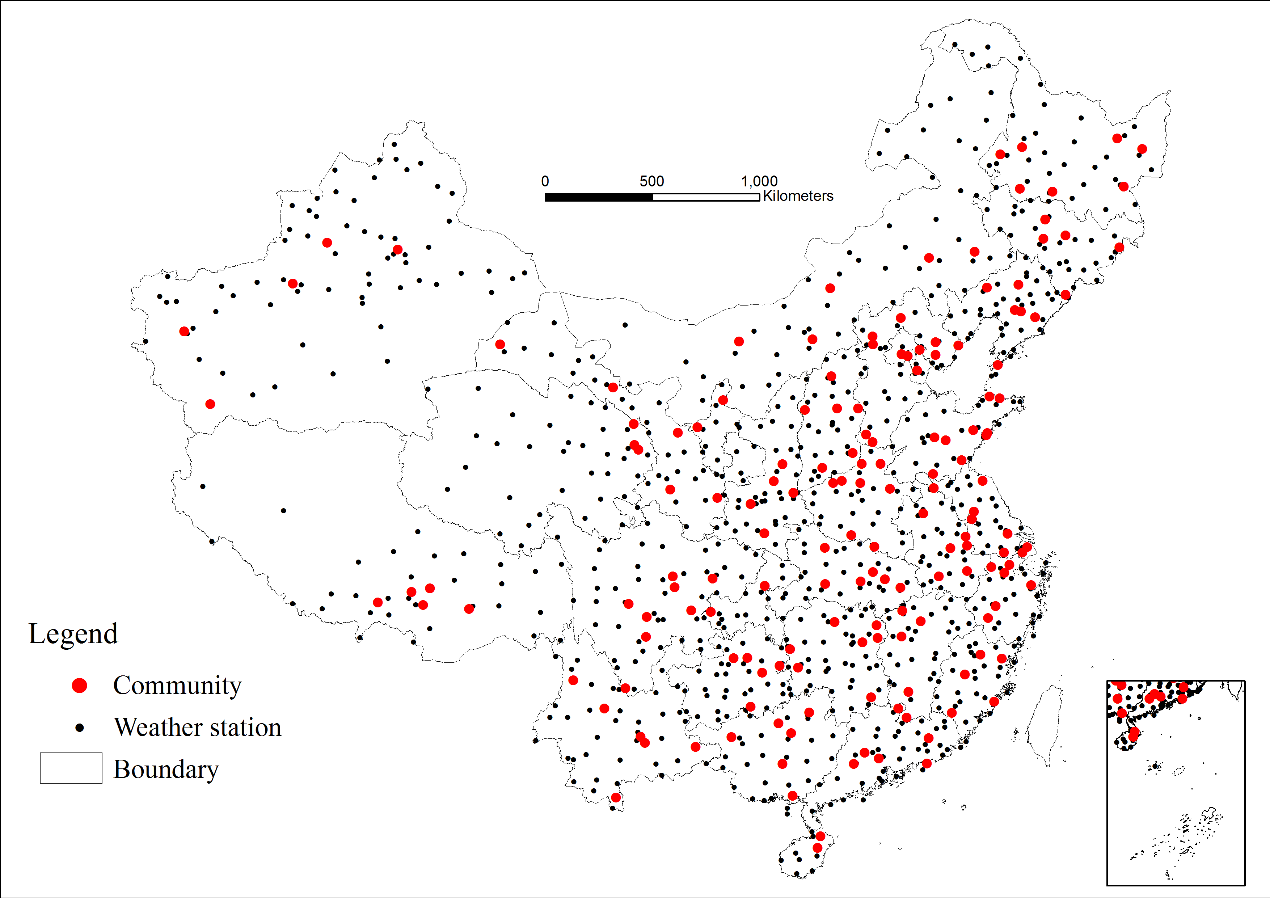


**Supplementary Fig. 1** Locations of the 161 disease surveillance points (red points) in China included in the study. (a) These surveillance points were categorized as the North, the Northwest, the Northeast, the Central, the East, the South and the Southwest; (b) black dots denote the distribution of 839 weather stations.


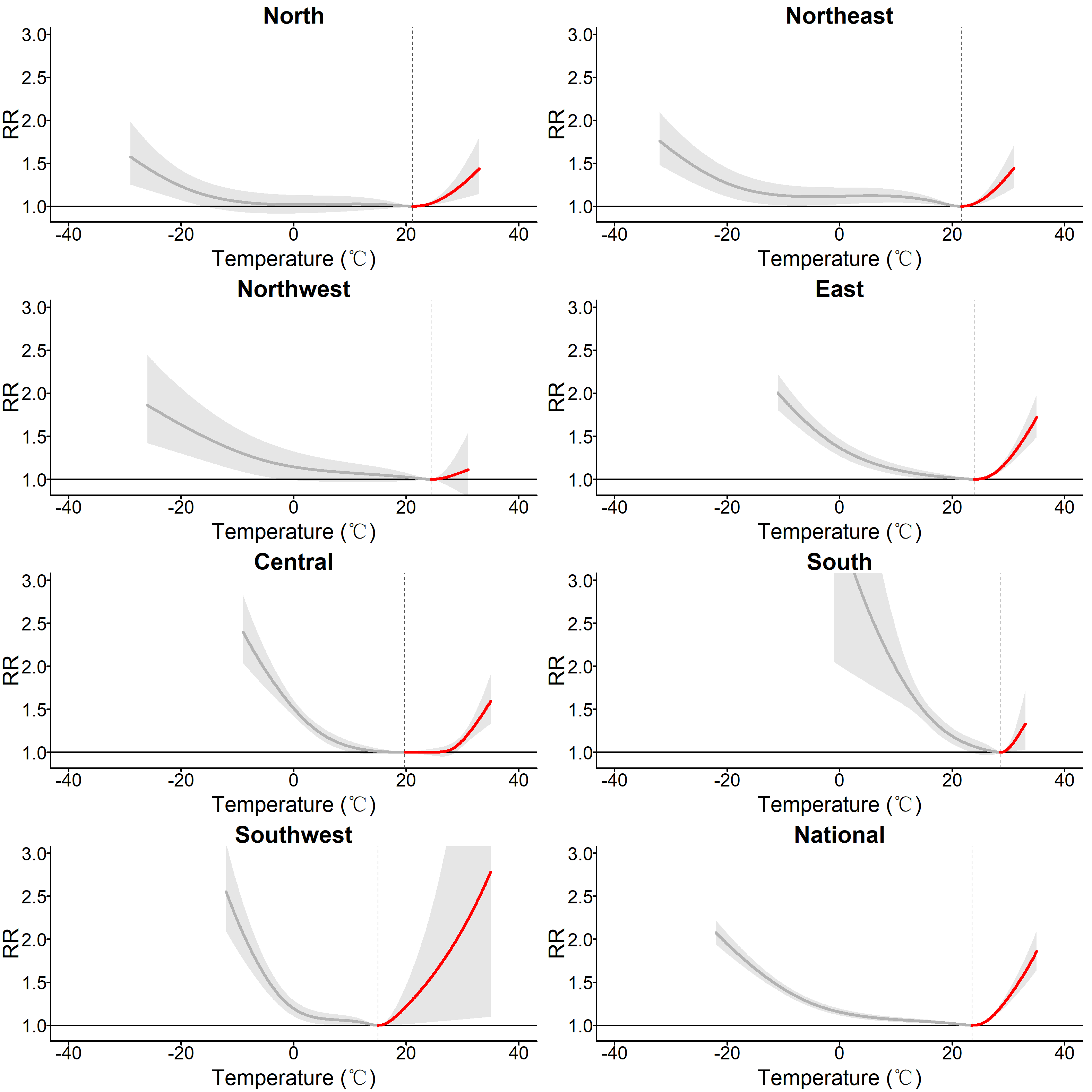


**Supplementary Fig. 2** The cumulative association between temperature and mortality across lag 0-14 days in regions. The red lines denote the relative risk (RR) of high temperature relative to the minimum mortality temperature (the vertical lines denote); the shaded areas represent the 95%CI. The stratified analysis was performed to establish the region-specific temperature-mortality association. Firstly, a distributed lag non-linear model was used to estimate the district/county-specific temperature-mortality association with 14 days of lag in each region adjusted for time trends and day of the week. Then, we separately pooled the temperature-mortality associations across districts/counties in each region by the multivariate meta-analysis.


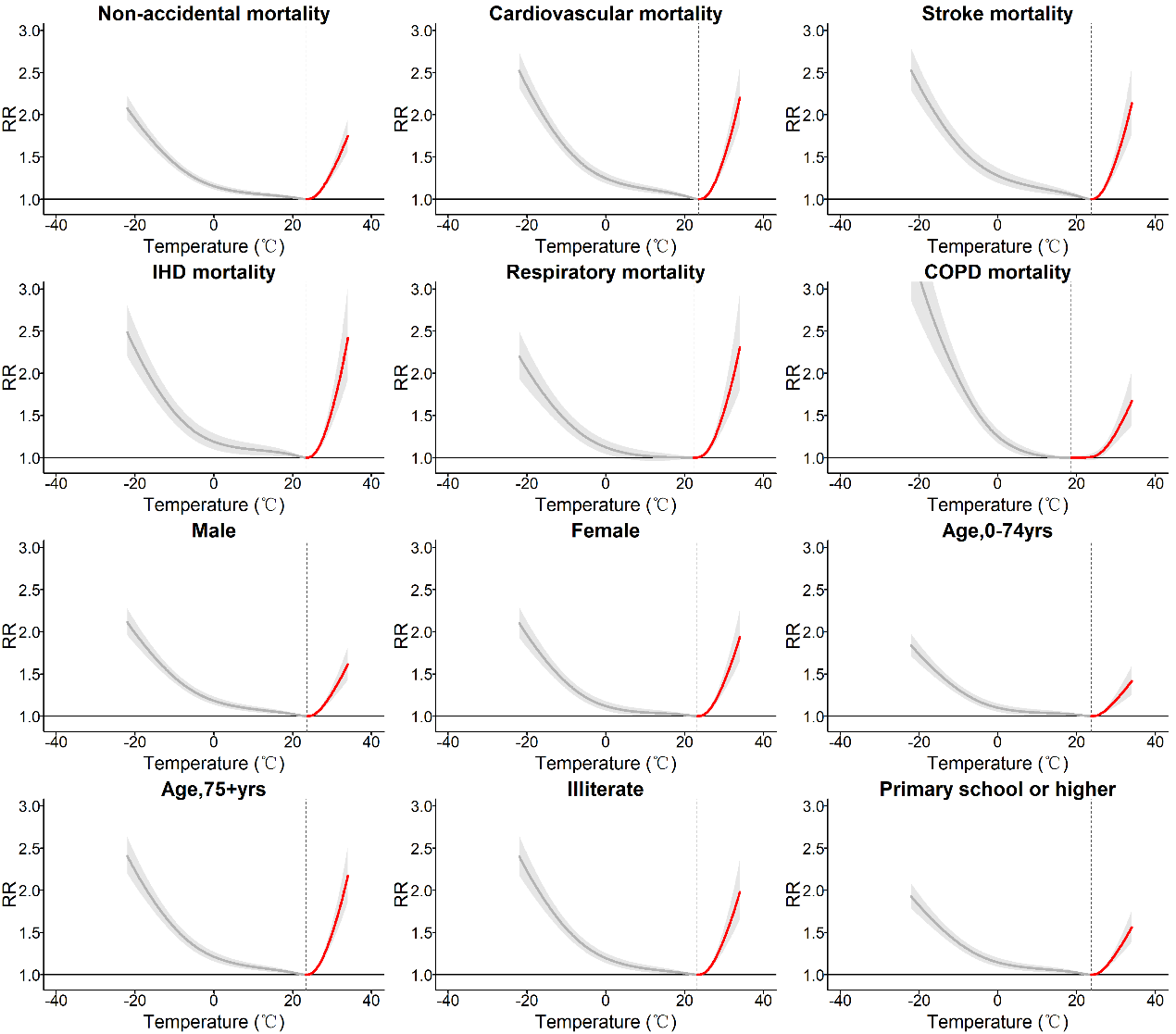


**Supplementary Fig. 3** The cumulative association between temperature and mortality across lag 0-14 days by individual characteristics. The red lines denote the relative risk (RR) of high temperature relative to the minimum mortality temperature (the vertical lines denote); the shaded areas represent the 95%CI. A distributed lag non-linear model was used to estimate district/county-specific temperature-mortality association with 14 days of lag adjusted for time trends and day of the week, which were pooled in a multivariate meta-analysis.


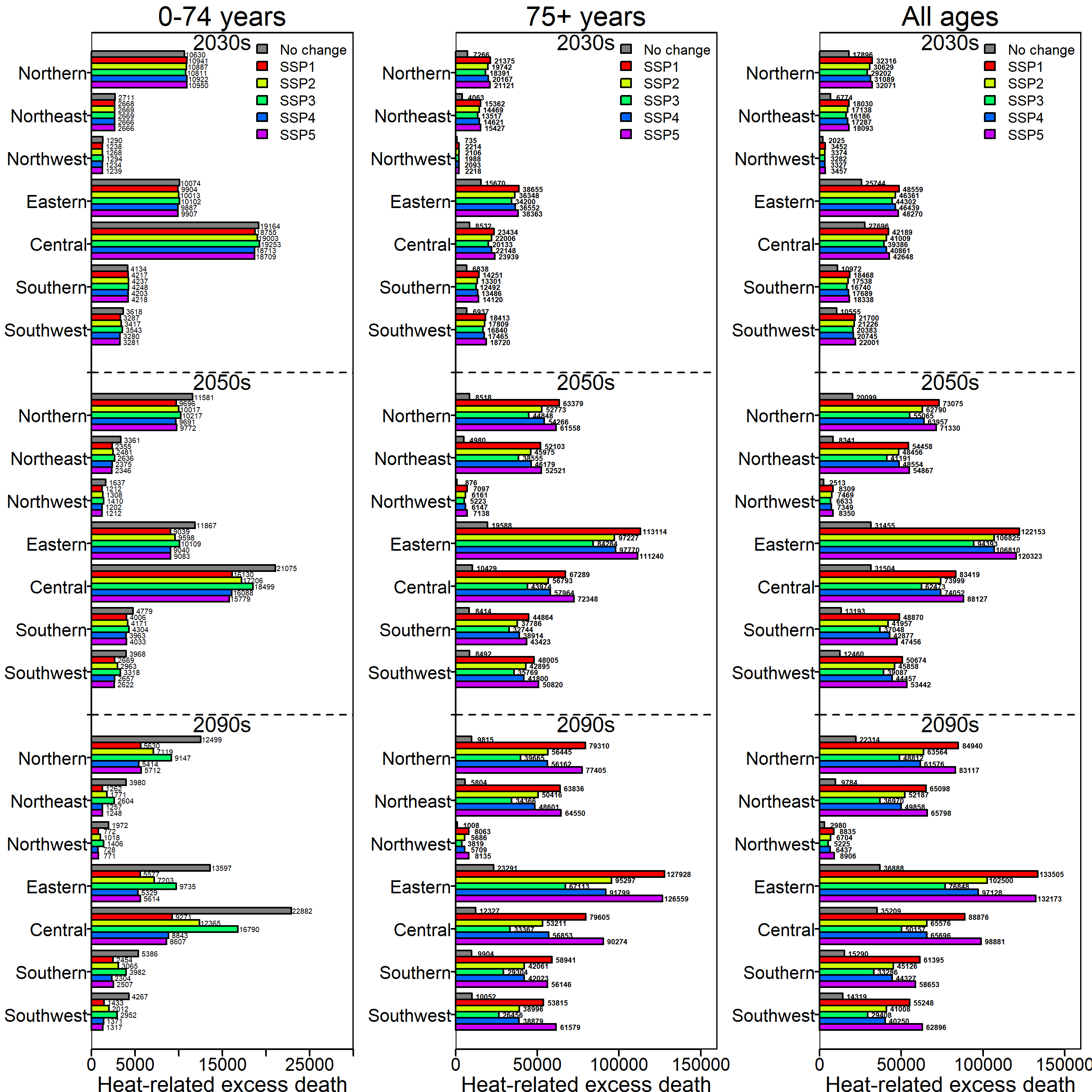


**Supplementary Fig. 4** The heat-related attributable number of deaths for different age groups under six population scenarios (no change, SSP1, SSP2, SSP3, SSP4 and SSP5) under RCP4.5 by regions in China.


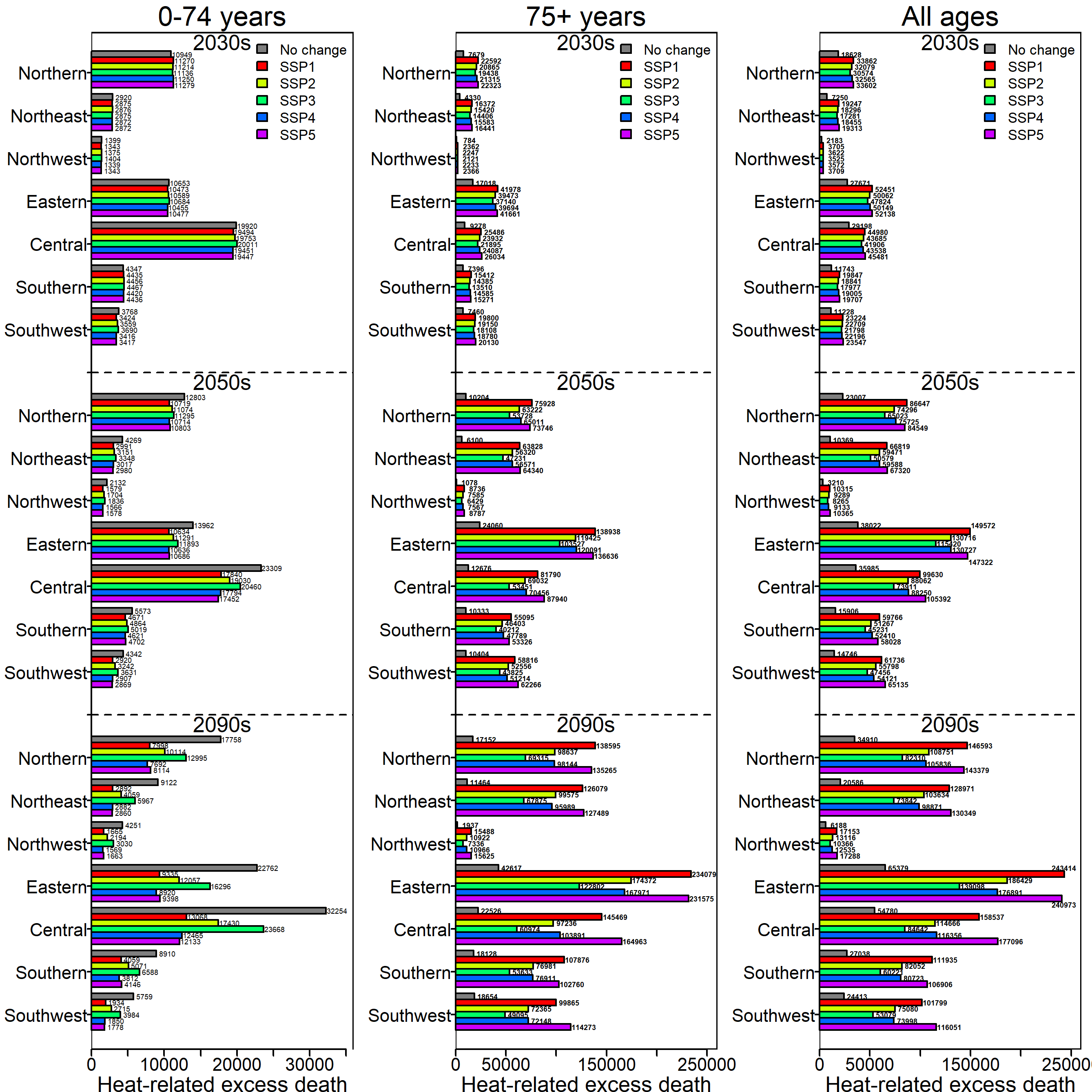


**Supplementary Fig. 5** The heat-related attributable number of deaths for different age groups under six population scenarios (no change, SSP1, SSP2, SSP3, SSP4 and SSP5) under RCP8.5 by region in China.


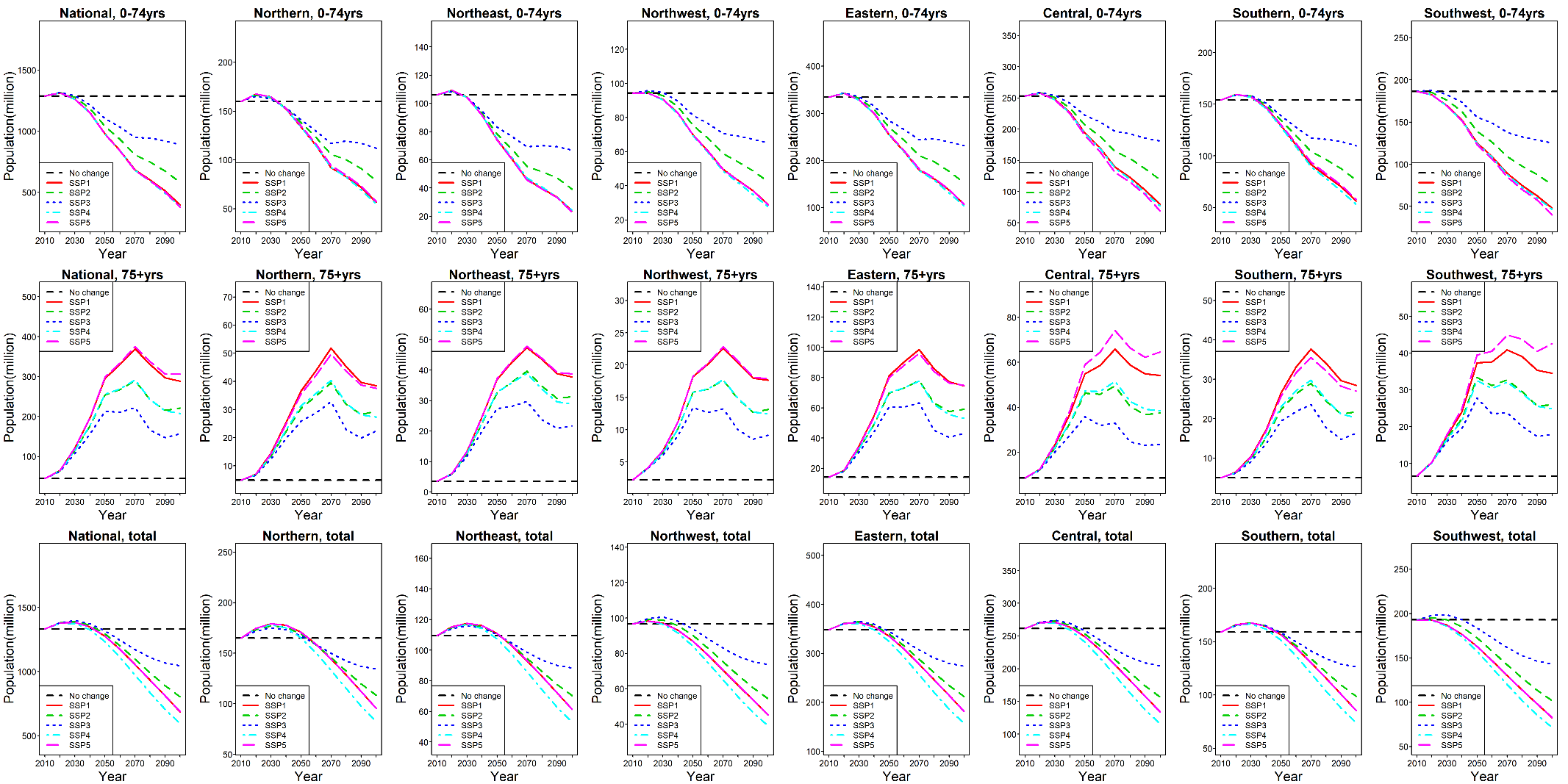


**Supplementary Fig. 6** The population projections for different age groups during 2010-2100 under six population scenarios (no change, SSP1, SSP2, SSP3, SPP4 and SSP5) by region in China.

**Supplementary References**

Arora, V. K., Scinocca, J. F., Boer, G. J., Christian, J. R., Denman, K. L., Flato, G. M., … Merryfield, W. J. (2011). Carbon emission limits required to satisfy future representative concentration pathways of greenhouse gases. *Geophysical Research Letters*, *38*(5). https://doi.org/https://doi.org/10.1029/2010GL046270

Baek, H.-J., Lee, J., Lee, H.-S., Hyun, Y.-K., Cho, C., Kwon, W.-T., … Byun, Y.-H. (2013). Climate change in the 21st century simulated by HadGEM2-AO under representative concentration pathways. *Asia-Pacific Journal of Atmospheric Sciences*, *49*(5), 603–618. https://doi.org/10.1007/s13143-013-0053-7

Bao, Y., Song, Z., & Qiao, F. (2020). FIO-ESM Version 2.0: Model Description and Evaluation. *Journal of Geophysical Research: Oceans*, *125*(6), e2019JC016036. https://doi.org/https://doi.org/10.1029/2019JC016036

Bentsen, M., Bethke, I., Debernard, J. B., Iversen, T., Kirkevåg, A., Seland, Ø., … Kristjánsson, J. E. (2013). The Norwegian Earth System Model, NorESM1-M – Part 1: Description and basic evaluation of the physical climate. *Geoscientific Model Development*, *6*(3), 687–720. https://doi.org/10.5194/gmd-6-687-2013

Dufresne, J.-L., Foujols, M.-A., Denvil, S., Caubel, A., Marti, O., Aumont, O., … Vuichard, N. (2013). Climate change projections using the IPSL-CM5 Earth System Model: from CMIP3 to CMIP5. *Climate Dynamics*, *40*(9), 2123–2165. https://doi.org/10.1007/s00382-012-1636-1

Dunne, J. P., John, J. G., Adcroft, A. J., Griffies, S. M., Hallberg, R. W., Shevliakova, E., … Zadeh, N. (2012). GFDL’s ESM2 Global Coupled Climate–Carbon Earth System Models. Part I: Physical Formulation and Baseline Simulation Characteristics. *Journal of Climate*, *25*(19), 6646–6665. https://doi.org/10.1175/JCLI-D-11-00560.1

Giorgetta, M. A., Jungclaus, J., Reick, C. H., Legutke, S., Bader, J., Böttinger, M., … Stevens, B. (2013). Climate and carbon cycle changes from 1850 to 2100 in MPI-ESM simulations for the Coupled Model Intercomparison Project phase 5. *Journal of Advances in Modeling Earth Systems*, *5*(3), 572–597. https://doi.org/https://doi.org/10.1002/jame.20038

Griffies, S. M., Winton, M., Donner, L. J., Horowitz, L. W., Downes, S. M., Farneti, R., … Zadeh, N. (2011). The GFDL CM3 Coupled Climate Model: Characteristics of the Ocean and Sea Ice Simulations. *Journal of Climate*, *24*(13), 3520–3544. https://doi.org/10.1175/2011JCLI3964.1

Hansen, J., Sato, M., Ruedy, R., Kharecha, P., Lacis, A., Miller, R., … Zhang, S. (2007). Climate simulations for 1880–2003 with GISS modelE. *Climate Dynamics*, *29*(7), 661–696. https://doi.org/10.1007/s00382-007-0255-8

Hazeleger, W., Wang, X., Severijns, C., Ştefănescu, S., Bintanja, R., Sterl, A., … van der Wiel, K. (2012). EC-Earth V2.2: description and validation of a new seamless earth system prediction model. *Climate Dynamics*, *39*(11), 2611–2629. https://doi.org/10.1007/s00382-011-1228-5

Ji, D., Wang, L., Feng, J., Wu, Q., Cheng, H., Zhang, Q., … Zhou, M. (2014). Description and basic evaluation of Beijing Normal University Earth System Model (BNU-ESM) version 1. *Geoscientific Model Development*, *7*(5), 2039–2064. https://doi.org/10.5194/gmd-7-2039-2014

Langehaug, H. R., Sandø, A. B., Årthun, M., & Ilıcak, M. (2019). Variability along the Atlantic water pathway in the forced Norwegian Earth System Model. *Climate Dynamics*, *52*(1), 1211–1230. https://doi.org/10.1007/s00382-018-4184-5

Leng, G., Tang, Q., & Rayburg, S. (2015). Climate change impacts on meteorological, agricultural and hydrological droughts in China. *Global and Planetary Change*, *126*, 23–34. https://doi.org/https://doi.org/10.1016/j.gloplacha.2015.01.003

Liu, X., Wu, T., Yang, S., Li, Q., Cheng, Y., Liang, X., … Nie, S. (2014). Relationships between interannual and intraseasonal variations of the Asian-western Pacific summer monsoon hindcasted by BCC_CSM1.1(m). *Advances in Atmospheric Sciences*, *31*(5), 1051–1064. https://doi.org/10.1007/s00376-014-3192-6

Meehl, G. A., Washington, W. M., Arblaster, J. M., Hu, A., Teng, H., Kay, J. E., … Strand, W. G. (2013). Climate Change Projections in CESM1(CAM5) Compared to CCSM4. *Journal of Climate*, *26*(17), 6287–6308. https://doi.org/10.1175/JCLI-D-12-00572.1

Meehl, G. A., Washington, W. M., Arblaster, J. M., Hu, A., Teng, H., Tebaldi, C., … White III, J. B. (2012). Climate System Response to External Forcings and Climate Change Projections in CCSM4. *Journal of Climate*, *25*(11), 3661–3683. https://doi.org/10.1175/JCLI-D-11-00240.1

Rotstayn, L. D., Jeffrey, S. J., Collier, M. A., Dravitzki, S. M., Hirst, A. C., Syktus, J. I., & Wong, K. K. (2012). Aerosol- and greenhouse gas-induced changes in summer rainfall and circulation in the Australasian region: a study using single-forcing climate simulations. *Atmospheric Chemistry and Physics*, *12*(14), 6377–6404. https://doi.org/10.5194/acp-12-6377-2012

Scoccimarro, E., Gualdi, S., Bellucci, A., Sanna, A., Giuseppe Fogli, P., Manzini, E., … Navarra, A. (2011). Effects of Tropical Cyclones on Ocean Heat Transport in a High-Resolution Coupled General Circulation Model. *Journal of Climate*, *24*(16), 4368–4384. https://doi.org/10.1175/2011JCLI4104.1

Volodin, E. M., Dianskii, N. A., & Gusev, A. V. (2010). Simulating present-day climate with the INMCM4.0 coupled model of the atmospheric and oceanic general circulations. *Izvestiya, Atmospheric and Oceanic Physics*, *46*(4), 414–431. https://doi.org/10.1134/S000143381004002X

Watanabe, M., Suzuki, T., O’ishi, R., Komuro, Y., Watanabe, S., Emori, S., … Kimoto, M. (2010). Improved Climate Simulation by MIROC5: Mean States, Variability, and Climate Sensitivity. *Journal of Climate*, *23*(23), 6312–6335. https://doi.org/10.1175/2010JCLI3679.1

Watanabe, S., Hajima, T., Sudo, K., Nagashima, T., Takemura, T., Okajima, H., … Kawamiya, M. (2011). MIROC-ESM 2010: model description and basic results of CMIP5-20c3m experiments. *Geoscientific Model Development*, *4*(4), 845–872. https://doi.org/10.5194/gmd-4-845-2011

Weare, B. C., Cagnazzo, C., Fogli, P. G., Manzini, E., & Navarra, A. (2012). Madden-Julian Oscillation in a climate model with a well-resolved stratosphere. *Journal of Geophysical Research: Atmospheres*, *117*(D1). https://doi.org/https://doi.org/10.1029/2011JD016247

Wu, T., Li, W., Ji, J., Xin, X., Li, L., Wang, Z., … Zhang, J. (2013). Global carbon budgets simulated by the Beijing Climate Center Climate System Model for the last century. *Journal of Geophysical Research: Atmospheres*, *118*(10), 4326–4347. https://doi.org/https://doi.org/10.1002/jgrd.50320

YUKIMOTO, S., ADACHI, Y., HOSAKA, M., SAKAMI, T., YOSHIMURA, H., HIRABARA, M., … KITOH, A. (2012). A New Global Climate Model of the Meteorological Research Institute: MRI-CGCM3 &mdash;Model Description and Basic Performance&mdash; *Journal of the Meteorological Society of Japan. Ser. II*, *90A*, 23–64. https://doi.org/10.2151/jmsj.2012-A02
