# Supplementary material for: Projecting heat-related excess mortality under climate change scenarios in China
Source: Nat Commun. 2021 Feb 15;12:1039. doi: 10.1038/s41467-021-21305-1 (PMC7884743; doi:10.1038/s41467-021-21305-1)
Supplement: Supplementary file 3 — Description of Additional Supplementary Files [file 41467_2021_21305_MOESM3_ESM.pdf]

Description of Additional Supplementary Files

File Name: Supplementary Data 1
Description: The annual mortality rate (per million populations) and annual mean temperature (oC) in 161 Chinese disease surveillance points.

File Name: Supplementary Data 2
Description: The temporal trends in annual projected temperature under two RCP scenarios in China.

File Name: Supplementary Data 3.
Description: The trends in heat-related excess mortality by period, cause and individual characteristic in China, assuming no adaptation or population changes.

File Name: Supplementary Data 4
Description: The heat-related attributable number of deaths by period, age and population scenario in China.
