# Supplementary material for: Projecting heat-related excess mortality under climate change scenarios in China
Source: Nat Commun. 2021 Feb 15;12:1039. doi: 10.1038/s41467-021-21305-1 (PMC7884743; doi:10.1038/s41467-021-21305-1)
Supplement: Supplementary file 8 — Reporting Summary [file 41467_2021_21305_MOESM8_ESM.pdf]

nature research

Corresponding author(s): Jun Yang & Qiyong Liu

Last updated by author(s): Oct 14, 2020

Reporting Summary

Nature Research wishes to improve the reproducibility of the work that we publish. This form provides structure for consistency and transparency in reporting. For further information on Nature Research policies, see our Editorial Policies and the Editorial Policy Checklist.

Statistics

For all statistical analyses, confirm that the following items are present in the figure legend, table legend, main text, or Methods section.

n/a	Confirmed	
☐	☒	The exact sample size (n) for each experimental group/condition, given as a discrete number and unit of measurement
☒	☐	A statement on whether measurements were taken from distinct samples or whether the same sample was measured repeatedly
☐	☒	The statistical test(s) used AND whether they are one- or two-sided<br>*Only common tests should be described solely by name; describe more complex techniques in the Methods section.*
☐	☒	A description of all covariates tested
☒	☐	A description of any assumptions or corrections, such as tests of normality and adjustment for multiple comparisons
☐	☒	A full description of the statistical parameters including central tendency (e.g. means) or other basic estimates (e.g. regression coefficient) AND variation (e.g. standard deviation) or associated estimates of uncertainty (e.g. confidence intervals)
☐	☒	For null hypothesis testing, the test statistic (e.g. F, t, r) with confidence intervals, effect sizes, degrees of freedom and P value noted<br>*Give P values as exact values whenever suitable.*
☒	☐	For Bayesian analysis, information on the choice of priors and Markov chain Monte Carlo settings
☒	☐	For hierarchical and complex designs, identification of the appropriate level for tests and full reporting of outcomes
☒	☐	Estimates of effect sizes (e.g. Cohen's d, Pearson's r), indicating how they were calculated

Our web collection on statistics for biologists contains articles on many of the points above.

Software and code

Policy information about availability of computer code

Data collection	All the data preparations and analyses were conducted through the R Foundation for Statistical Computing, version 3.5.1. Maps were generated using ArcGIS software, version 10.3.
Data analysis	All the data preparations and analyses were conducted through the R Foundation for Statistical Computing, version 3.5.1. Maps were generated using ArcGIS software, version 10.3.

For manuscripts utilizing custom algorithms or software that are central to the research but not yet described in published literature, software must be made available to editors and reviewers. We strongly encourage code deposition in a community repository (e.g. GitHub). See the Nature Research guidelines for submitting code & software for further information.

Data

Policy information about availability of data

All manuscripts must include a data availability statement. This statement should provide the following information, where applicable:

- Accession codes, unique identifiers, or web links for publicly available datasets
- A list of figures that have associated raw data
- A description of any restrictions on data availability

The dataset generated and analyzed during this study are available (with some institutional limitations) from the corresponding authors upon reasonable request. The mortality data can be obtained from the Chinese Center for Disease Control and Prevention under reasonable request. Unfortunately, we do not have the rights to publish the mortality data. Historical temperature data are available at http://data.cma.cn/. The NWAI-WG statistical downscaled CMIP5 GCMs data for the observed climate sites from China Meteorological Data Service Center are available at https://www.agrivy.com/. Gridded population projections of China for five SSPs are available at www.cgd.ucar.edu/iam/modeling/spatial-population-scenarios.html. Air pollution data can be obtained through reasonable request.

Field-specific reporting

Please select the one below that is the best fit for your research. If you are not sure, read the appropriate sections before making your selection.

☐ Life sciences ☐ Behavioural & social sciences ☒ Ecological, evolutionary & environmental sciences

For a reference copy of the document with all sections, see nature.com/documents/nr-reporting-summary-flat.pdf

Ecological, evolutionary & environmental sciences study design

All studies must disclose on these points even when the disclosure is negative.

Study description	In this study, we quantified the excess mortality risk of future heat exposure by causes of death, regions and individual characteristics in 161 Chinese districts/counties under different future weather scenarios using 28 general climate models. We further assessed the influence of population aging on future heat-related excess mortality under five shared socioeconomic pathways to deepen our understanding of vulnerability characteristics of climate change.
Research sample	All deaths occurred in the selected 161 districts/counties during 2007-2013 were included in our study. Totally, there were 2,742,717 non-accidental deaths, 1,274,558 cardiovascular deaths, 670,017 stroke deaths, 431,814 IHD deaths, 397,738 respiratory deaths and 302,125 COPD deaths
Sampling strategy	In order to ensure the generalization of our results to the whole country, we included mortality data from all surveillance points (161 districts/counties) during 2007-2013 in the Disease Surveillance Points system (DSPs) in China. DSPs was established using the multi-level random sampling strategy, including the first level of seven geographic regions (Northeast, Northern, Eastern, Southern, Southwest Northwest and Central areas) and three municipalities (Beijing, Tianjin and Shanghai), the second level of urban and rural, and further classification of rural sites into four socio economic strata and urban sites into three strata according to the population size. Then, the probability proportionate to population size sampling method was used to select the samples at different level.
Data collection	Daily mortality in 161 communities were collected from the Disease Surveillance Points system of Chinese Center for Disease Control and Prevention; daily temperature data (minimum, maximum and mean temperatures) from 839 weather stations during 2007-2013 were firstly collected from the China Meteorological Data Service Center; projected daily minimal and maximal temperatures in each community during 1971-2099 were collected from datasets of the 5th Coupled Model Inter-comparison Project phase 5; gridded population projections of China during 2010-2100 under five SSPs at 1km×1km resolution were extracted from the SSP spatial population scenario database (www.cgd.ucar.edu/iam/modeling/spatial-population-scenarios.html). Air pollution was predicted 2011-2013 were predicted by the modified community multiscale air quality (CMAQ) model. These data were collected by Jun Yang, Maigeng Zhou, Qiyong Liu, Peng Yin, De Li Liu, and Zhoupeng Ren.
Timing and spatial scale	Daily mortality were obtained from 161 districts/counties in Disease Surveillance Points system during 2007-2013; daily temperature during 2007-2013 were interpolated into 1km×1km; population projections of China during 2010-2100 under five SSPs at 1km×1km resolution; the projected temperature during 1971-2100 from 28 general circulation model simulations ranged from 0.7o (lat.) × 0.8o (lon.) to 3.7o × 3.78o; air pollution were predicted at a resolution of 36 km×36 km during 2011-2013. All the gridded data were aggregated for each district or county according to its boundary.
Data exclusions	No data were excluded from the analyses.
Reproducibility	All attempts to repeat the experiment were successful.
Randomization	We collected daily mortality data in 161 districts/counties (64 city districts in urban areas and 97 counties in rural areas) from the nationwide Disease Surveillance Points system (DSPs), which was established using the multi-stage random sampling strategy. But randomization was not applicable to the death case in each district or county.
Blinding	This study is a time-series design. Blinding was not applicable to the daily mortality data in this study. But considering the ethical issues, we did not collect the private information of each death case, such as identification card, name and address.

Did the study involve field work? ☐ Yes ☒ No

Reporting for specific materials, systems and methods

We require information from authors about some types of materials, experimental systems and methods used in many studies. Here, indicate whether each material, system or method listed is relevant to your study. If you are not sure if a list item applies to your research, read the appropriate section before selecting a response.

Materials & experimental systems

n/a	Involved in the study
☒	☐ Antibodies
☒	☐ Eukaryotic cell lines
☒	☐ Palaeontology and archaeology
☒	☐ Animals and other organisms
☒	☐ Human research participants
☒	☐ Clinical data
☒	☐ Dual use research of concern

Methods

n/a	Involved in the study
☒	☐ ChIP-seq
☒	☐ Flow cytometry
☒	☐ MRI-based neuroimaging
